# Characterization and genomic analysis of the highly virulent *Acinetobacter baumannii* ST1791 strain dominating in Anhui, China

**DOI:** 10.1128/aac.01262-24

**Published:** 2024-12-06

**Authors:** Zhien He, Yi Huang, Wei Li, Huanhuan Zhang, Ruobing Cao, Md Roushan Ali, Yuanyuan Dai, Huaiwei Lu, Wanying Wang, Qiuhong Niu, Baolin Sun, Yujie Li

**Affiliations:** 1 Department of Oncology, The First Affiliated Hospital of University of Science and Technology of China, Division of Life Sciences and Medicine, University of Science and Technology of China 612146 , Hefei, Anhui, China; 2 Department of Cancer Epigenetics Program, The First Affiliated Hospital of University of Science and Technology of China 117556 , Hefei, Anhui, China; 3 Department of Clinical Laboratory, the First Affiliated Hospital of University of Science and Technology of China, Division of Life Sciences and Medicine, University of Science and Technology of China 612146 , Hefei, Anhui, China; 4 School of Life Science, Nanyang Normal University 71072 , Nanyang, Henan, China; Universita degli studi di roma La Sapienza, Rome, Italy

**Keywords:** ST1791, whole genome sequencing, CC92, *Acinetobacter baumannii*

## Abstract

The multidrug-resistant *Acinetobacter baumannii* clonal complex 92 is spreading worldwide due to its high-frequency gene mutation and recombination, posing a significant threat to global medical and health safety. Between November 2021 and April 2022, a total of 132 clinical *A. baumannii* isolates were collected from a tertiary hospital in China. Their growth ability and virulence of these isolates were assessed using growth curve analyses and the *Galleria mellonella* infection model. The genetic characteristics of the isolates were further examined through whole-genome sequencing. ST1791^O^/ST2^P^ isolates represented the largest proportion of isolates in our collection and exhibited the highest growth rate and strongest virulence among all sequence types (STs) analyzed. Whole-genome sequences from 14,159 clinical isolates were collected from the National Center for Biotechnology Information database, and only nine ST1791^O^/ST2^P^ isolates were detected. Comparative genomic analysis revealed that ST1791^O^/ST2^P^ carried 11 unique genes, 5 of which were located within the capsular polysaccharide synthesis (*cps*) gene cluster. Single nucleotide polymorphisms (SNPs) between ST1791^O^/ST2^P^ and other isolates were primarily found in the cps gene cluster. Among the other isolates, ST195^O^/ST2^P^ and ST208^O^/ST2^P^ exhibited the smallest SNP differences from ST1791^O^/ST2^P^, while ST195^O^/ST2^P^ and ST1486^O^/ST2^P^ had high homology. The ST1791^O^/ST2^P^ strain in Anhui, China, displayed significant homology with ST195^O^/ST2^P^, ST208^O^/ST2^P^, and ST1486^O^/ST2^P^ isolates. Compared to other isolates in this study, ST1791^O^/ST2^P^ exhibited strong growth ability and virulence. Therefore, preventing the further spread of ST1791^O^/ST2^P^ should be a top public health priority.

## INTRODUCTION

*Acinetobacter baumannii* is an opportunistic Gram-negative pathogen that frequently causes severe infections, such as pneumonia and bloodstream infections, particularly in immunocompromised hosts (1). *A. baumannii* was previously regarded as a low-risk microorganism. However, with the growing number of reported infections caused by *A. baumannii*, it is now considered a harmful pathogen that poses a serious threat to public health (2).

Most clinical *A. baumannii* isolates from different countries and regions belong to international clones I, II, and III. International clone II typically accounts for the majority of strains (3–5). According to two international schemes for multilocus sequence typing (MLST) of *A. baumannii* (Bartual scheme, also known as the Oxford scheme [6] and Pasteur scheme [7]), these international clones can be further categorized into multiple clonal complexes (CCs). CC92^O^/CC2^P^ is the most commonly reported CC in international clone II and is the most widespread an abundant CC of *A. baumannii* globally (8). CC92^O^/CC2^P^ has been reported in Europe, North America, Asia, Africa, South America, and Oceania, with high detection rates in various regions across these continents (9–15). Compared to other CCs, CC92^O^/CC2^P^ demonstrates higher biofilm formation ability (16), stronger fitness (17), and higher rates of antibiotic resistance (18). However, the detailed mechanisms remain to be further investigated (8).

ST208^O^/ST2^P^ *A. baumannii*, belonging to CC92^O^/CC2^P^, has become the most commonly detected ST worldwide approximately 30 years after its first isolation in Australia (19). It is the most prevalent type in CC92^O^/CC2^P^ and the most common *A. baumannii* strain (20).

ST1791^O^/ST2^P^ also belongs to CC92^O^/CC2^P^ (21), but there have been no reports of a large-scale outbreak associated with this strain. Prior to this study, ST1791 had not been detected in the molecular epidemiology study of *A. baumannii* in Anhui Province, China (22, 23). Between November 2021 and April 2022, multidrug-resistant (MDR) *A. baumannii* isolates were collected from a tertiary hospital in Hefei, China. The results showed that 43 isolates were ST1791^O^/ST2^P^ (hereinafter referred to as ST1791), while nine isolates were ST208^O^/ST2^P^. This study marks the first report of a large-scale occurrence of ST1791. To deepen our understanding of ST1791, this study characterized its basic phenotypic properties and analyzed its genetic features and uniqueness through comparative genomic analysis.

## RESULTS

### ST1791 dominated the collected isolates

**Fig 1:**
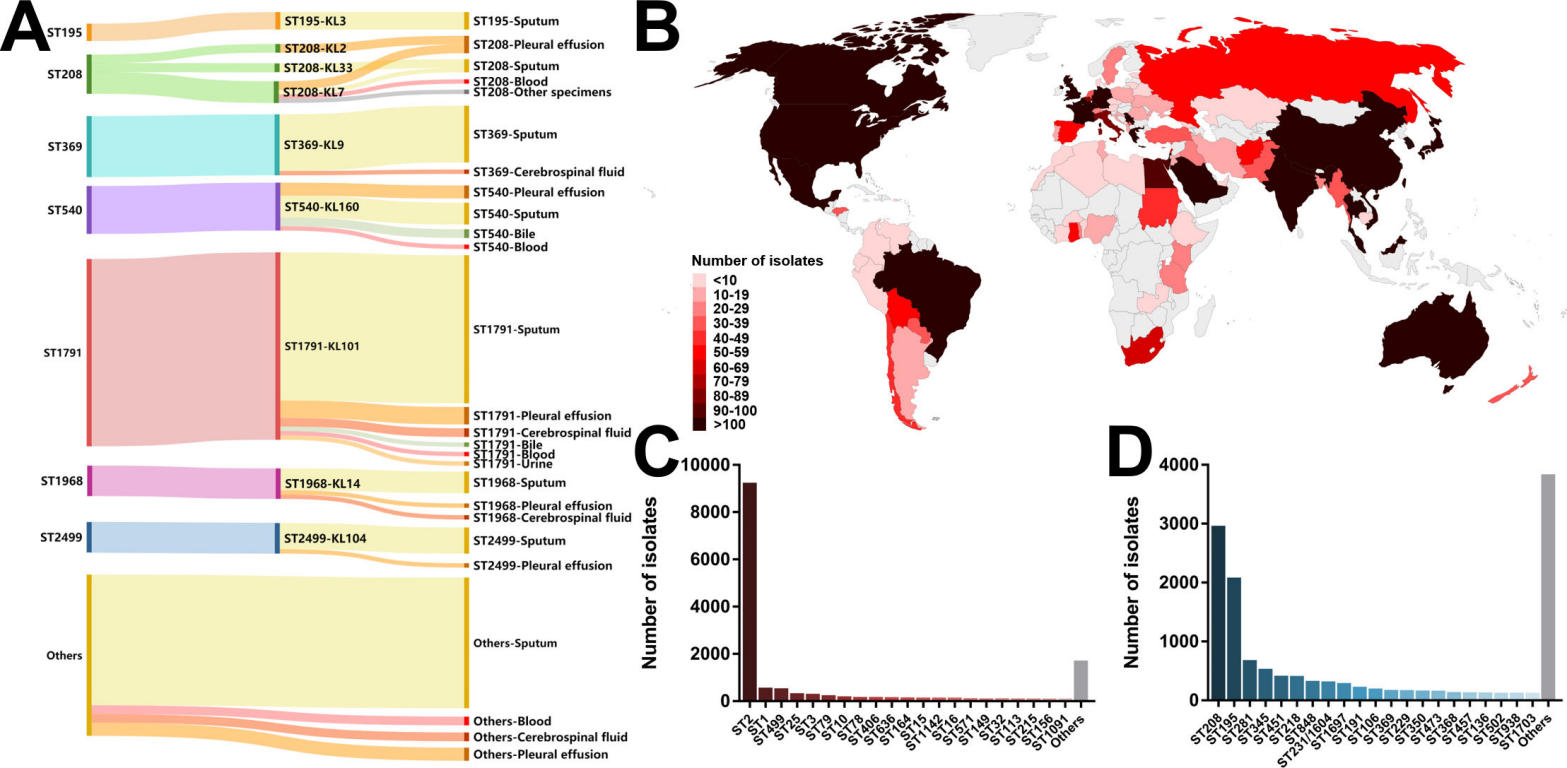
Overview of the basic properties of the isolates collected in this study and the distribution of clinical *A. baumannii* isolates in the National Center for Biotechnology Information (NCBI) database. (**A**) Summary of MLST, K-types, and the sources of the collected isolates. (**B**) Global distribution map of clinical *A. baumannii* isolates in the NCBI database; the depth of color indicates the number of isolates. (**C**) Number of different ST isolates in the NCBI database, detected using the Pasteur scheme. The quantities are arranged from left to right from most to least. (**D**) Number of different ST isolates in the NCBI database, detected using the Oxford scheme. The quantities are arranged from left to right from most to least.

Of the 132 isolates, the majority were recovered from sputum (*n* = 102; Fig. 1A) and some from pleural effusion (*n* = 16), cerebrospinal fluid (*n* = 6), blood (*n* = 5), and bile (*n* = 3; Table S1). All isolates were resistant to ciprofloxacin (Table S2). A total of 130 isolates (98.48%) were resistant to carbapenems. According to the Oxford scheme, the samples consisted of 43 ST1791^O^/ST2^P^ (32.58%), 14 ST369^O^/ST2^P^ (10.61%), 11 ST540^O^/ST2^P^ (8.33%), 9 ST208^O^/ST2^P^ (6.82%), 4 ST195^O^/ST2^P^ (3.03%), 7 ST1968^O^/ST2^P^ (5.30%), 7 ST2499^O^/ST2^P^ (5.30%), and 37 other ST-type isolates (28.03%; Table S3). The capsular type (K-type) of all ST1791 isolates was KL101, while ST195 isolates had KL3, ST369 isolates had KL9, ST540 isolates had KL160, ST1968 isolates had KL14, and ST2499 isolates had KL104. ST208 isolates displayed greater diversity (KL2 = 2, KL7 = 5, and KL33 = 2). No significant differences in antimicrobial susceptibility testing were observed between isolates from different STs.

To investigate the global prevalence of ST1791, genomic data for all 14,159 clinical *A. baumannii* isolates from the National Center for Biotechnology Information (NCBI) database were collected up to 31 December 2022 (Fig. S1). The United States and China reported the highest numbers of isolates (6,821 and 2,003, respectively), while other countries and regions had significantly fewer isolates (Fig. 1B). According to the Pasteur scheme, the STs of these isolates were relatively simple (Fig. 1C). ST2 accounted for most isolates (9,171, 64.77%). In contrast, the STs were more diverse according to the Oxford scheme (Fig. 1D). The most common types were ST208 (2,934, 20.72%) and ST195 (2,055, 14.51%). In addition, 450 STs remained, which is 87 more STs than identified in the Pasteur scheme. However, only nine isolates were classified as ST1791. All of these isolates were from China with the K-type of KL101 and were collected in 2018 or 2019. The most common K-types included KL2 (2,708, 19.13%), KL3 (2,239, 15.81%), KL9 (1,084, 7.66%), and KL22 (850, 6.00%). Only 25 isolates were identified as KL101 (Fig. S2). These results suggest that ST1791 was not frequently detected prior to this analysis.

### ST1791 has a faster growth rate and enhanced virulence

**Fig 2:**
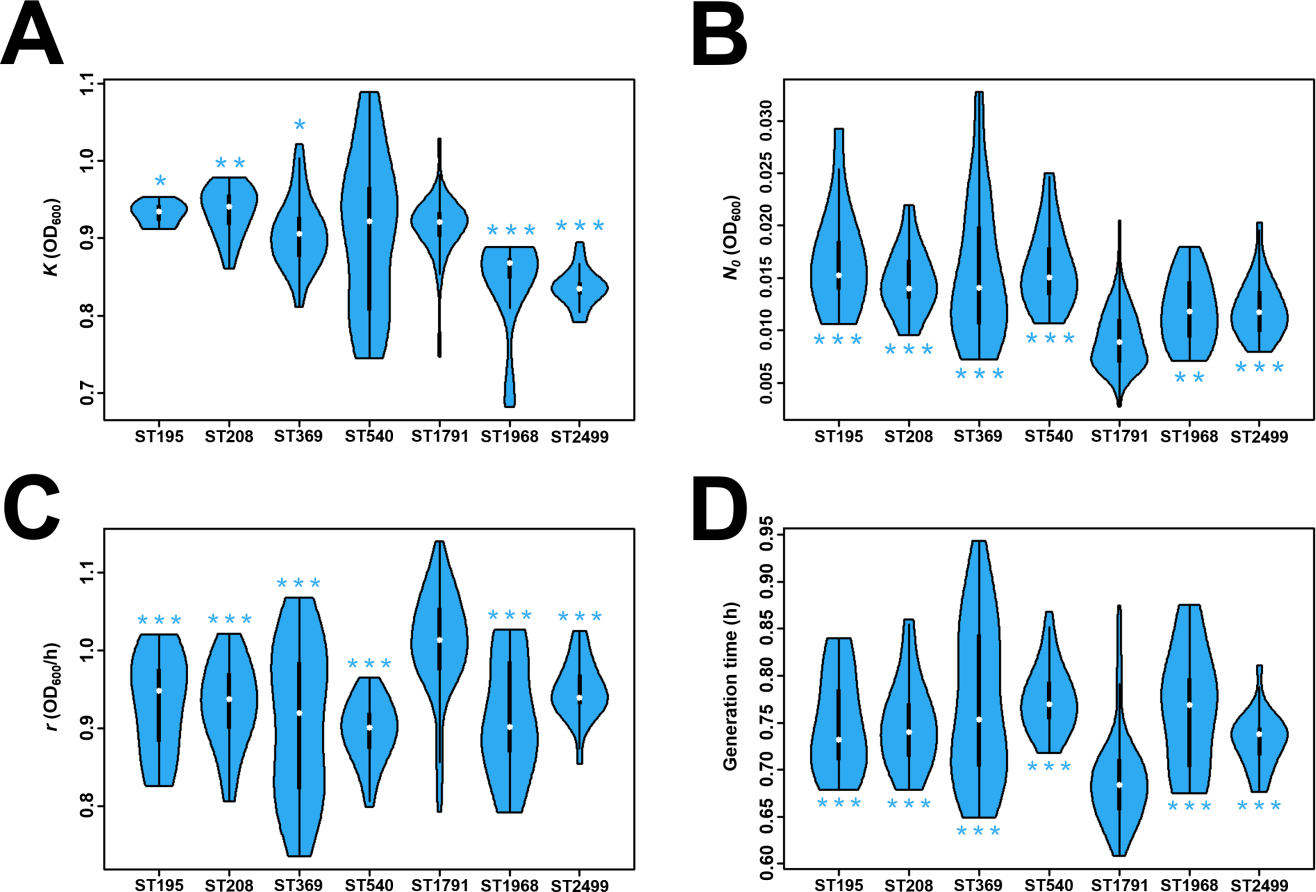
ST1791 exhibits the fastest growth rate and shortest generation time. Comparison of growth curve parameters among the isolates obtained from the growth curve mathematical model. Growth curves were performed in triplicate, with strain ATCC 19606 used as a control. Panels **A**, **B**, **C**, and **D** show violin plots of maximum population size (***K***), initial population size (***N_0_***), population growth rate (***R***), and population generation time (t_gene) among the different isolates, respectively. Statistical significance for other ST isolates was compared with ST1791. *P < 0.05; **P < 0.01; ***P < 0.001; and ****P < 0.0001.

**Fig 3:**
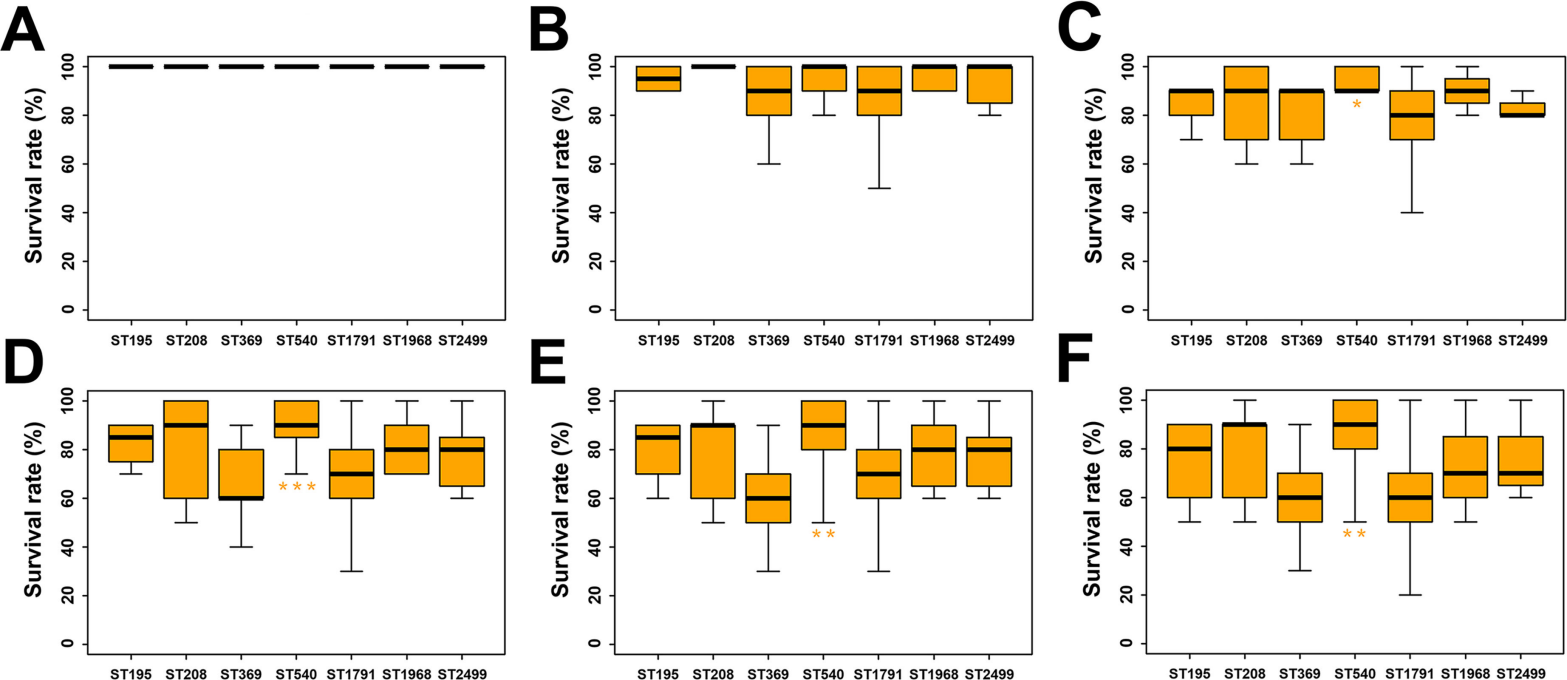
ST1791 isolates showed high virulence in the *G. mellonella* infection model. The *G. mellonella* infection model was conducted using strain ATCC 19606 as a control and was performed in triplicate (1 × 10^6^ CFU). Panels **A**, **B**, **C**, **D**, **E**, and **F** represent the survival rates of *G. mellonella* at 12 h, 24 h, 36 h, 48 h, 60 h, and 72 h after infection with *A. baumannii*, respectively. Statistical significance for other ST isolates was compared with ST1791. *P < 0.05; **P < 0.01; ***P < 0.001; and ****P < 0.0001.

How does ST1791 differ from other CC92 isolates? Growth curve experiments were performed on ST1791 and other CC92 isolates, including ST195, ST208, ST369, ST540, ST1791, ST1968, and ST2499, which did not belong to CC92 but exhibited similarities to it (a total of 95 isolates; see Fig. S3 to S5). The parameters *K* (the maximum population size in a specific environment), *N_0_* (the population size at the beginning of the growth curve), *r* (the growth rate without imposing restrictions on the population), and the generation time were obtained according to the mathematical model of the growth curve (Fig. S6). ST1791 had the smallest *N_0_* (0.009 ± 0.003 OD_600_), the fastest *r* (1.010 ± 0.067 OD_600_/h), and the shortest generation time (0.690 ± 0.050 h; Fig. 2). These results suggest that the ST1791 population has the greatest growth capacity. The *Galleria mellonella* infection model was used to assess the differences in virulence between ST1791 and other isolates (Fig. S7 to S9). At 36, 48, 60, and 72 hours, the lethality of ST1791 and ST369 in *G. mellonella* was significantly higher than that of ST540. However, no significant difference was observed between the other ST isolates and ST540 (Fig. 3). Therefore, ST540 isolates were less virulent in *G*. *mellonella* than ST1791 and ST369 (Table 1).

**TABLE 1:** Basic clinical information and characteristics of the *A. baumannii* isolates collected in this study

Variable	ST1791	ST195	ST208	ST369	ST540	ST1968	ST2499
Patient information							
Age (years, mean ± SD)	65.95 ± 14.21	60.00 ± 20.41	51.22 ± 17.78	60.07 ± 6.47	56.82 ± 18.10	61.71 ± 20.27	58.43 ± 20.38
Source							
Bile	1 (2.33%)	0 (0.00%)	0 (0.00%)	0 (0.00%)	2 (18.18%)	0 (0.00%)	0 (0.00%)
Blood	1 (2.33%)	0 (0.00%)	1 (11.11%)	0 (0.00%)	1 (9.09%)	0 (0.00%)	0 (0.00%)
Cerebrospinal fluid	2 (4.65%)	0 (0.00%)	0 (0.00%)	1 (7.14%)	0 (0.00%)	1 (14.29%)	0 (0.00%)
Pleural effusion	4 (9.30%)	0 (0.00%)	4 (44.44%)	0 (0.00%)	3 (27.27%)	1 (14.29%)	1 (14.29%)
Sputum	34 (79.07%)	4 (100.00%)	3 (33.33%)	13 (92.86%)	5 (45.45%)	5 (71.43%)	6 (85.71%)
Urine	1 (2.33%)	0 (0.00%)	0 (0.00%)	0 (0.00%)	0 (0.00%)	0 (0.00%)	0 (0.00%)
Other specimens	0 (0.00%)	0 (0.00%)	1 (11.11%)	0 (0.00%)	0 (0.00%)	0 (0.00%)	0 (0.00%)
Growth curve							
*K* (OD_600_, mean ± SD)	0.916 ± 0.039	0.932 ± 0.014	0.934 ± 0.032	0.906 ± 0.045	0.907 ± 0.098	0.841 ± 0.064	0.836 ± 0.024
*N_o_* (OD_600_, mean ± SD)	0.009 ± 0.003	0.017 ± 0.005	0.015 ± 0.003	0.016 ± 0.007	0.016 ± 0.004	0.012 ± 0.003	0.012 ± 0.003
*r* (OD_600_/h, mean ± SD)	1.010 ± 0.067	0.931 ± 0.064	0.930 ± 0.056	0.909 ± 0.096	0.896 ± 0.040	0.912 ± 0.074	0.950 ± 0.038
*t_gen* (h, mean ± SD)	0.690 ± 0.050	0.748 ± 0.053	0.748 ± 0.046	0.771 ± 0.083	0.775 ± 0.036	0.765 ± 0.062	0.730 ± 0.030
*G. mellonella* infection model							
12h survival rate (%, mean ± SD )	96.51 ± 8.97	100.00 ± 0.00	96.67 ± 7.07	98.57 ± 3.63	99.10 ± 3.02	100.00 ± 0.00	98.57 ± 3.78
24h survival rate (%, mean ± SD )	86.28 ± 14.64	95.00 ± 5.77	92.22 ± 15.63	87.86 ± 11.88	94.55 ± 6.88	95.71 ± 5.35	92.86 ± 9.51
36h survival rate (%, mean ± SD )	78.37 ± 15.73	85.00 ± 10.00	86.67 ± 15.81	81.43 ± 10.99	90.00 ± 13.42	82.86 ± 24.30	82.86 ± 9.51
48h survival rate (%, mean ± SD )	67.44 ± 17.74	82.50 ± 9.57	78.89 ± 21.47	65.00 ± 13.45	88.18 ± 16.01	74.29 ± 26.30	77.14 ± 14.96
60h survival rate (%, mean ± SD )	65.81 ± 19.42	80.00 ± 14.14	77.78 ± 20.48	60.00 ± 16.17	87.27 ± 16.18	72.86 ± 27.52	77.14 ± 14.96
72h survival rate (%, mean ± SD )	60.93 ± 19.50	75.00 ± 19.15	75.56 ± 20.07	59.29 ± 17.30	84.55 ± 18.64	68.57 ± 27.95	75.71 ± 15.12
Single nucleotide polymorphism amount (compared with ST1791, mean ± SD)	408.04 ± 399.61	1,431.41 ± 189.62	2,396.14 ± 708.69	2,812.77 ± 1017.54	2,815.69 ± 355.86	1,635.47 ± 270.19	9,346.47 ± 108.21

### Comparative genomic analysis of ST1791 isolates

**Fig 4:**
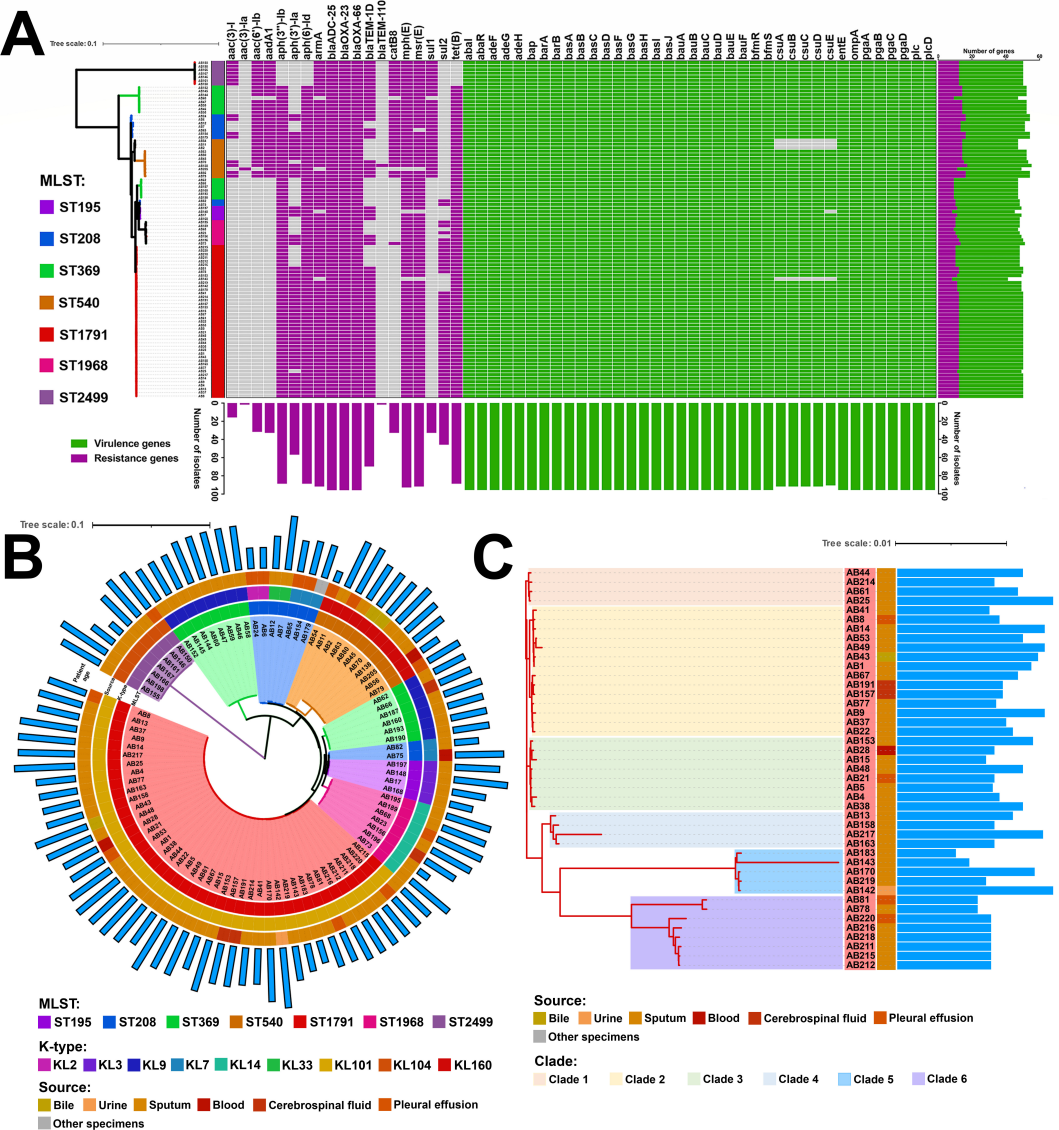
Phylogenetic tree and gene distribution heatmap of CC92 isolates collected in this study. (**A**) Distribution of drug resistance and virulence genes among CC92 isolates.The histogram below the figure represents the numbers of detected drug resistance and virulence genes, while the histogram on the right indicates the number of drug resistance and virulence genes carried by each strain. Carrying resistance genes are shown in purple, and carrying virulence factors are in green. The scale bar represents the number of nucleotide substitutions per site. (**B**) Circular phylogenetic tree of the CC92 isolates. From the inner ring to the outer ring, the data display the STs, K-types, source, and patient age for each isolates. (**C**) Phylogenetic tree of ST1791 isolates collected in this study. From the inner ring to the outer ring, the data depict the source and patient age for each isolates.

**Fig 5:**
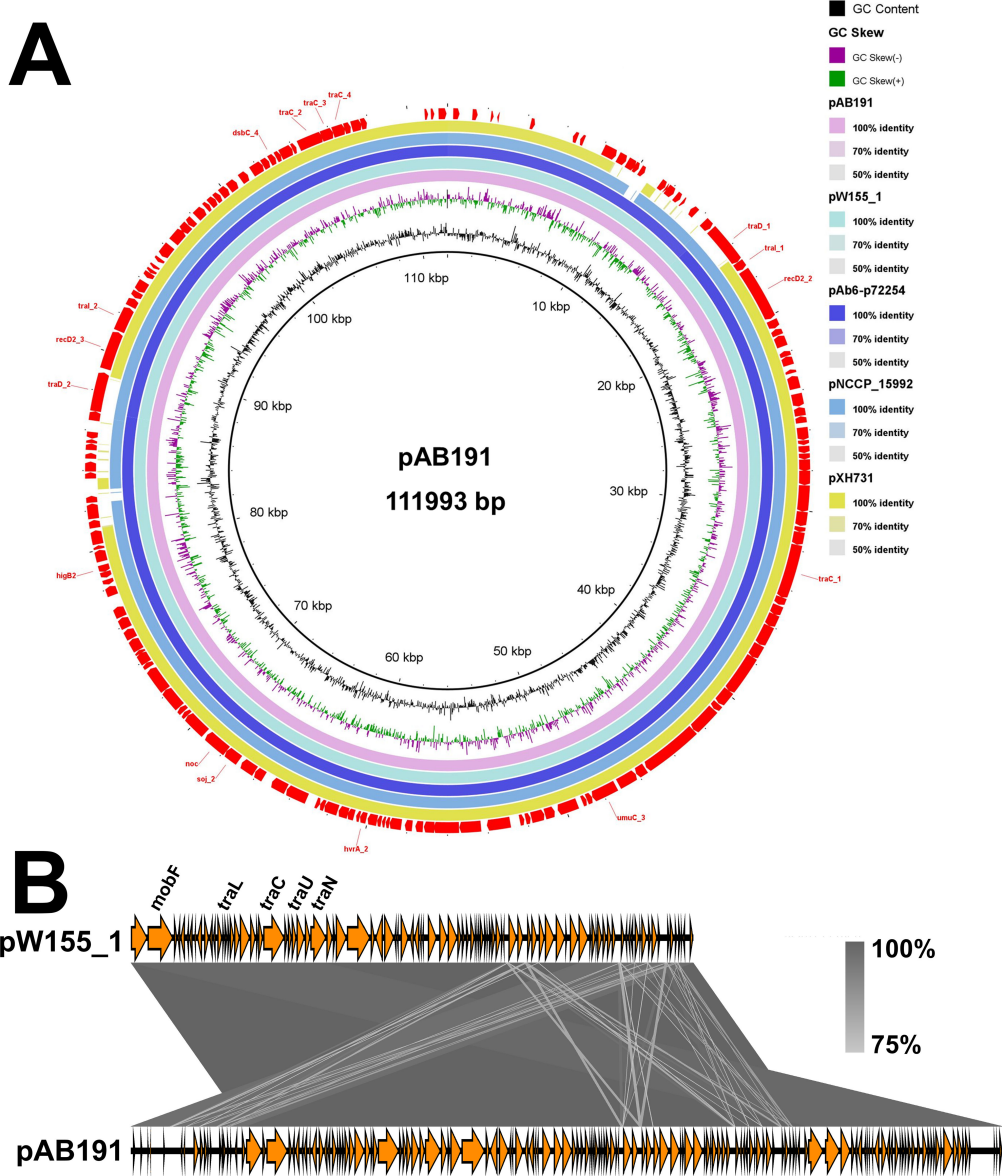
Plasmid sequence of pAB191 and its alignment map. (**A**) Alignment of plasmid pAB191, plasmid pW155_1 (GenBank accession no. CP163040), plasmid pAb6-72245_2 (GenBank accession no. CP159580), plasmid pNCCP_15992 (GenBank accession no. CP099787), and plasmid pXH731 (GenBank accession no. CP021322), generated using the BLAST Ring Image Generator 0.95 (24). (**B**) Alignment map of plasmid pAB191 and pW155_1, generated using Easyfig 2.2.5 (25).

Whole-genome sequencing was performed on the isolates mentioned above. The results showed that all isolates carried the carbapenem resistance genes *bla*_OXA-23_ and *bla*_OXA-66_ (Fig. 4A). Phylogenetic analysis classified each MLST branch into different subclades based on their respective K-types (Fig. 4B). All ST1791 isolates formed a distinct clade. The unrooted tree revealed that the ST1791 isolates had the shortest genetic distance among all ST195 and some ST208 isolates (AB75 and AB82; Fig. S10). Phylogenetic analysis divided the ST1791 isolates into six clades. Clades 1–3 were closely related, whereas clades 4–6 were more distantly related to clades other than themselves (Fig. 4C). Third-generation sequencing results showed that ST1791 carried a 111,993 bp plasmid, designated pAB191 (Fig. 5A). Plasmid pAB191 exhibited 99.99% similarity and 100% coverage with plasmid pW155_1 (CP163040), which is found in ST136^O^/ST2^P^ strain W155 isolated from Kunming, China (Fig. 5B). Although no transposable elements were identified in pAB191, it was a combination of two pW155_1 plasmids.

To further clarify the genomic characteristics of ST1791, a pan-genome analysis was conducted on ST1791 and 223 CC92 *A*. *baumannii* strains with complete genome sequences available in the NCBI database. In addition to the plasmid pAB191, ST1791 was also found to carry 11 unique genes compared to other CC92 strains (Table S4). These 11 genes included five genes in the *cps* synthesis gene cluster (DJJOKIHN_03865, DJJOKIHN_03866: *wzy*, DJJOKIHN_03867, DJJOKIHN_03868: *wbpI*, and DJJOKIHN_03869: *dapH*), one tRNA-Asp (gtc) encoding gene (DJJOKIHN_00644), one glutathione S-transferase family protein-encoding gene (DJJOKIHN_01478), and four genes with unknown function (DJJOKIHN_01396, DJJOKIHN_01397, DJJOKIHN_01401, and DJJOKIHN_01404). The proteins encoded by these four genes with unknown functions are all less than 100 amino acids long and are located near the bacterial nucleoid-associated protein *hupB* (gene number in the genome annotation is DJJOKIHN_01400). Additionally, there are no transposable elements in the surrounding genes.

**Fig 6:**
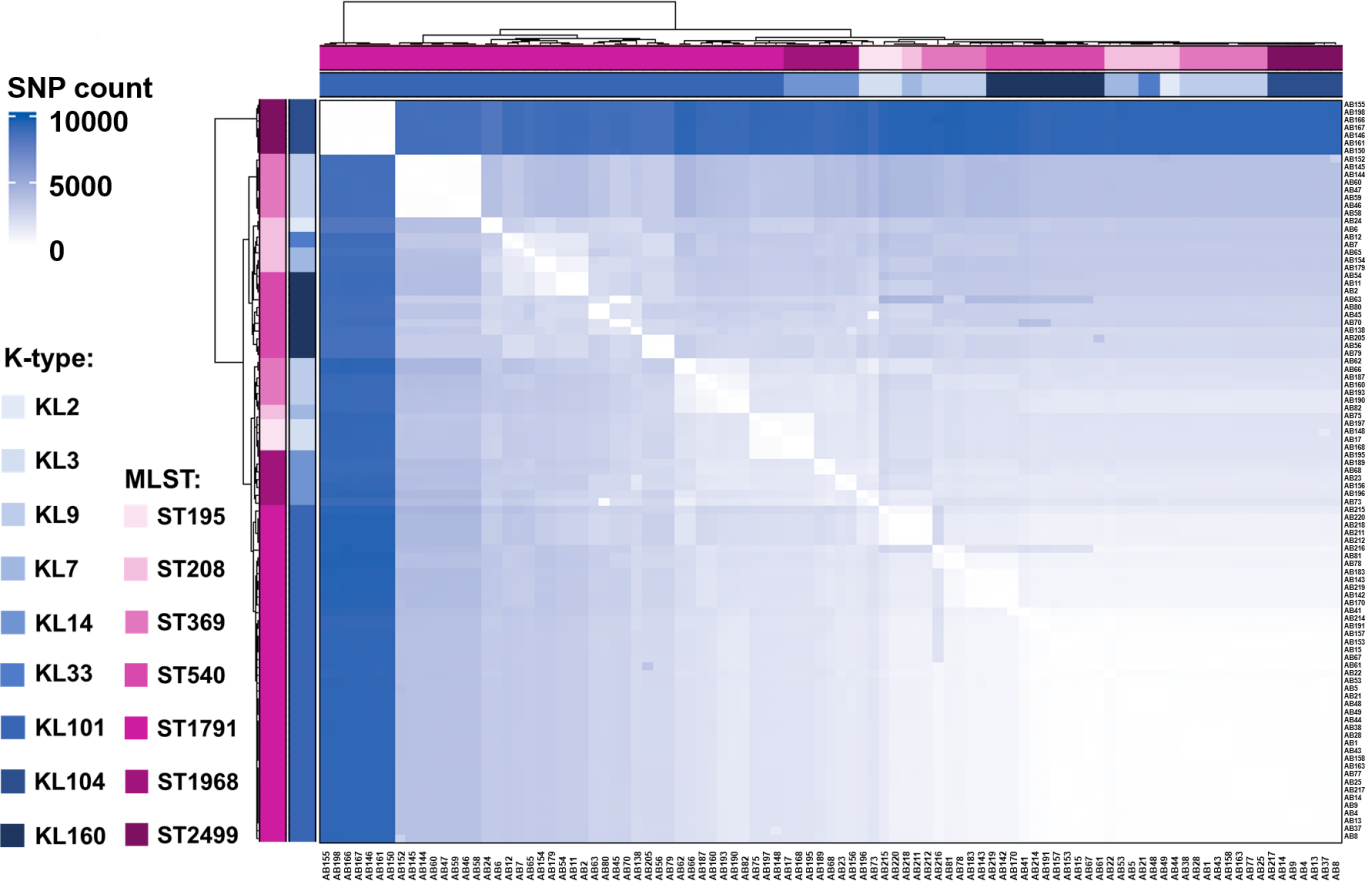
SNP differential distribution among the isolates collected in this study. The greater the number of SNPs among the isolates, the darker the color.

**Fig 7:**
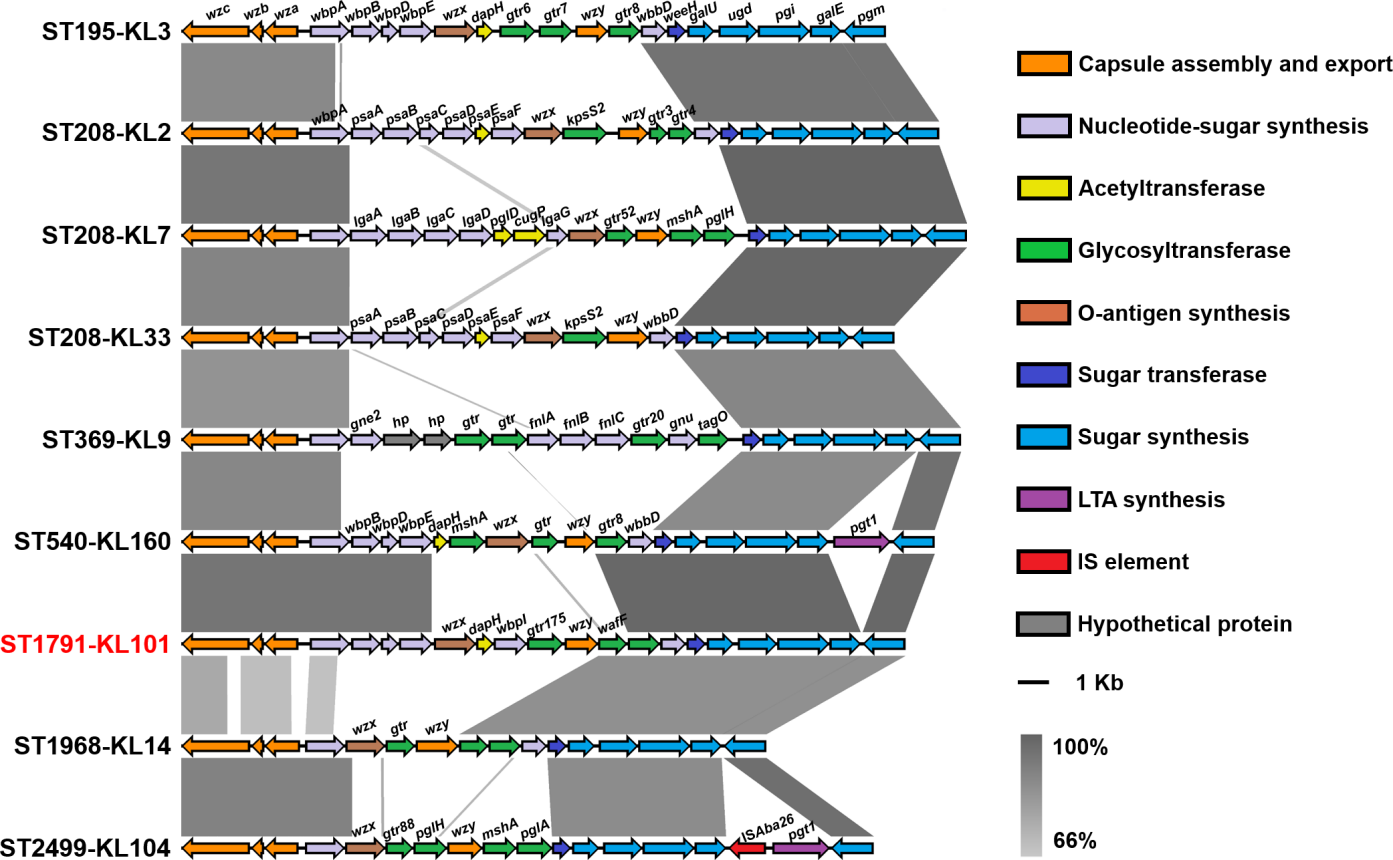
Sequence alignment of capsular polysaccharide synthesis (*cps*) gene clusters among different isolates. Different colors indicate various types of genes within the *cps* gene cluster.

To further elucidate the genomic characteristics of ST1791, single nucleotide polymorphisms (SNPs) were compared between ST1791 and other isolates (Fig. 6). The result showed that ST195 and ST1791 had the lowest number of SNPs (1,431.41 ± 189.62) in the whole genome, whereas ST2499 and ST1791 had the highest number of SNPs (9,346.47 ± 108.21; Table S5). There were also few SNP differences between the two ST208 isolates in the unrooted tree, which were grouped in the ST195 branch and ST1791 (AB75: 1,313.14 ± 180.84 and AB82: 1,286.81 ± 206.42). Most of these SNPs were concentrated in the *cps* gene cluster and its surrounding regions (such as *wzc*, *wzx*, *wzy*, *pgi*, and *galU*). Comparative analysis showed that *wzc-wzb-wza-wbpA*, located upstream of the *cps* gene cluster, and *weeH-galU-ugd-pgi-galE-pgm*, located downstream, were relatively conserved among different isolates (Fig. 7). Furthermore, *wbpI* was exclusively found in the *cps* gene cluster of ST1791-KL101.

### Missense mutations in *pmrB* cause polymyxin resistance in ST1791 *A. baumannii* isolate

Four polymyxin B-resistant isolates, including an ST1791 isolate designated AB191 (MIC = 8 µg/mL), were collected. AB191 belonged to ST1791-KL101, with a chromosome size of 4,075,106 bp, a total of 3,949 coding genes and a plasmid, pAB191 (Fig. S11). SNP differences between AB191 and other ST1791 isolates were analyzed, revealing that only one mutation was unique to AB191 (Fig. S12). The mutation occurred in PmrB, specifically, the valine at position 300 of PmrB was mutated to glutamic acid (c.899A > T, p. Glu300Val).

**Fig 8:**
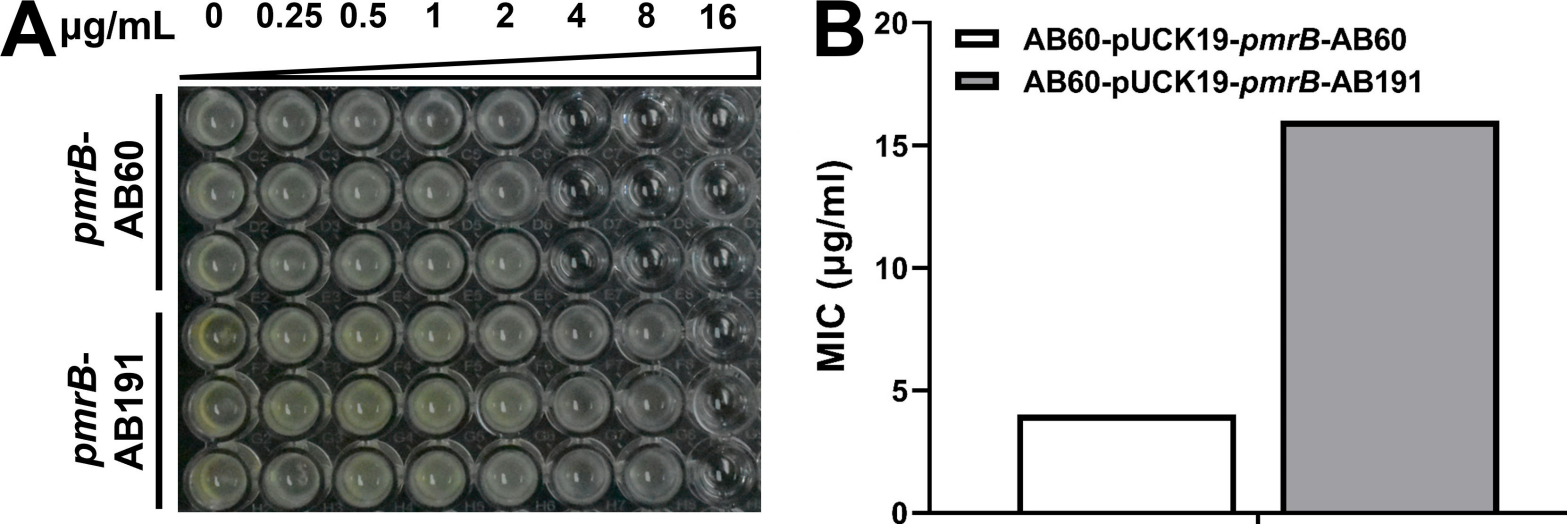
Missense mutations in *pmrB* result in resistance to polymyxin B in ST1791 *A. baumannii* AB191. (**A**) The polymyxin B MIC of AB60 carrying plasmids pUCK19-*pmrB*-AB60 and pUCK19-*pmrB*-AB191. The upper portion displays the concentration gradient of polymyxin B in microgram per milliliter. (**B**) The polymyxin B MIC of AB60 carrying plasmids with different genes.

To verify whether the *pmrB* mutation is relevant for polymyxin resistance, a method previously used to assess the hypermucoviscosity of the strain was used (26). This involved constructing strains carrying the predicted polymyxin resistance mutant and wild-type genes, along with the promoters of these genes expressed from constitutive expression plasmids (pUCK19-*pmrB*-AB191 carrying mutant *pmrB* of AB191 and pUCK19-*pmrB*-AB60 carrying wild-type *pmrB* of AB60). These plasmids were transformed into AB60, and the polymyxin B MIC was determined for AB60 carrying different plasmids. The results showed that the polymyxin B MIC of AB60 carrying pUCK19-*pmrB*-AB191 was 16 µg/mL, while that of AB60 carrying pUCK19-*pmrB*-AB60 was 4 µg/mL (Fig. 8A and B). This indicates that the *pmrB* mutation of AB191 significantly increases the resistance of the strain to polymyxin B. Therefore, the resistance of AB191 to polymyxin B primarily derives from the missense *pmrB* mutation.

### ST1791 has high homology with ST195 and other CC92 strains

**Fig 9:**
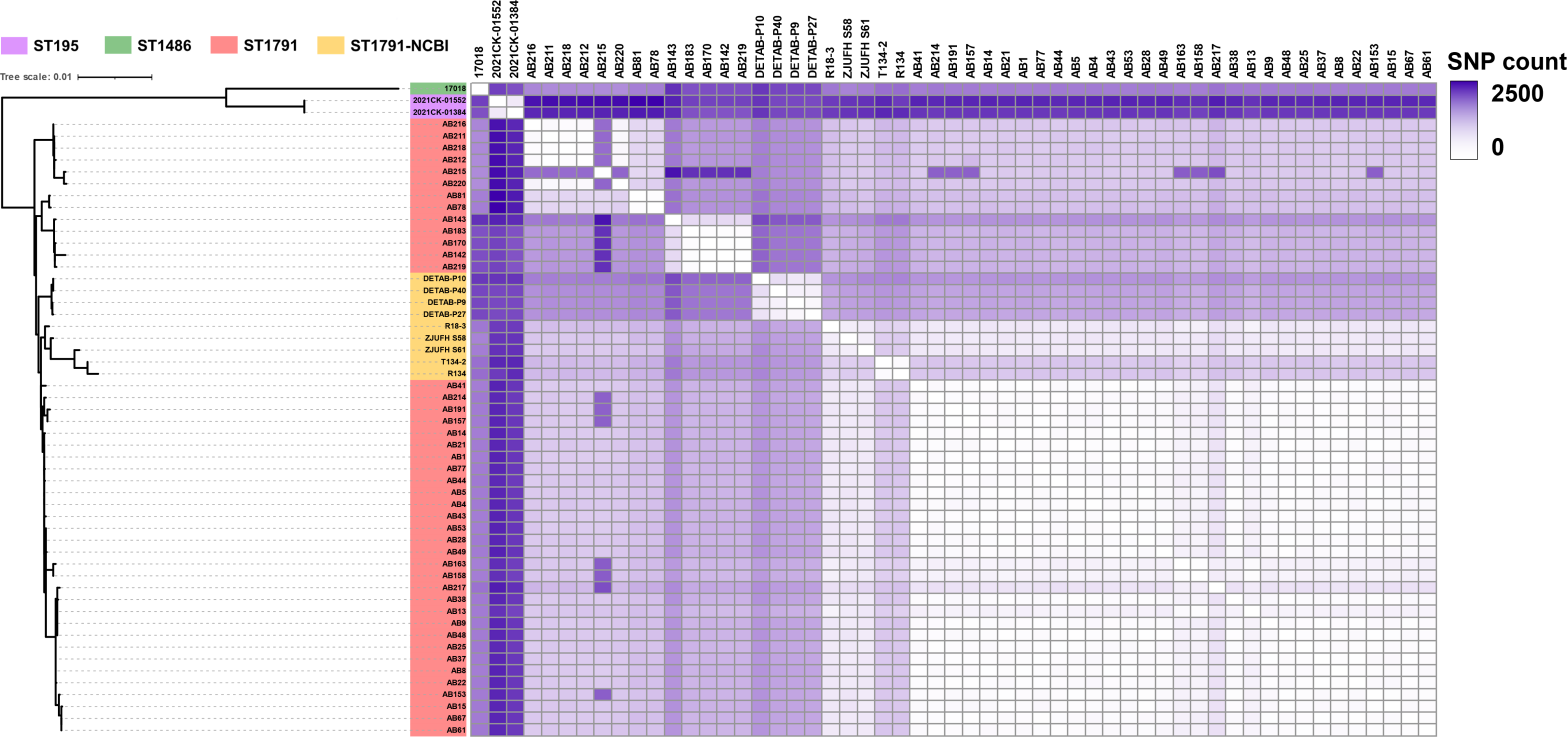
The ST1791 isolates in this study form a separate clade with strains from the NCBI database. In Fig. S15, this study’s ST1791 isolates and other strains from the NCBI database form a triangular phylogenetic tree representing a distinct clade. Purple indicates the ST195 strains, green represents the ST1486 strain, red denotes the ST1791 isolates collected in this study, and orange signifies the ST1791 strains from the NCBI database. The left side of the figure displays the phylogenetic tree formed by ST1791 in this study and the strains from the NCBI database. The right side of the figure shows a heat map of SNP differences between different strains. The greater the number of SNPs among the isolates, the darker the color.

To further explore the origin of ST1791, ATCC 19606 was used as the reference genome to construct a phylogenetic tree of 9,171 CC92 isolates from the NCBI database, along with the ST1791 isolates. However, due to the excessive number of strains and the uneven distribution of differences among them, minor differences in some strains were ignored. As a result, the collected ST1791 isolates, which were scattered across one of the branches, mainly consisted of strains ST195 and ST208 (Fig. S13). Further screening of 9,171 strains from the NCBI database was conducted. In the phylogenetic tree, the ST1791 isolates in this study and the 5,795 CC92 strains from the NCBI database formed a large clade together. Therefore, the genome of ST208 strain BM2333 (a strain not belonging to the same clade as ST1791; GenBank accession No. CP091328) was used as a reference genome, and a phylogenetic analysis was conducted between the ST1791 isolates collected in this study and these 5,795 strains (Fig. S14). The results showed that the ST1791 isolates collected in this study, the ST1791 strains from the NCBI database, two ST195 strains, and one ST1486 strain were classified into the same clade (Fig. S15). Information on this clade was extracted from the original phylogenetic tree file, and a triangular phylogenetic tree of this clade was reconstructed. In the phylogenetic tree, the ST1791 isolates in this study formed four major branches, while the ST1791 strains from the NCBI database formed two major branches. Additionally, the two ST195 strains (2021CK-01384 and 2021CK-01552, from the United States) and one ST1486 strain (17018, from China) formed one branch (Fig. 9). The SNP analysis indicated that the average SNP difference between the ST1791 strains in the NCBI database and those in this study was 746.04 ± 377.13. The average SNP difference between the two ST195 strains and the ST1791 isolates in this study was 2168.45 ± 156.34, while the average SNP difference between one ST1486 strain and the ST1791 isolates in this study was 1394.81 ± 196.90.

## DISCUSSION

Since its first report, CC92 has spread widely across the world, and dozens of STs have been detected (27). Among the CC92 populations, ST208 and ST195 are undoubtedly the most prevalent *A. baumannii* strains, as supported by the NCBI database. Although CC92 ST1791 *A. baumannii* was described in this study, it has rarely been reported. The first reports of ST1791 date back to 2020 in Jiangsu, China (21). ST1791 was also reported in China in 2023, but these findings did not receive significant attention (28). Among the 132 MDR *A. baumannii* isolates collected in this study, only four isolates were ST195, and 9 isolates were ST208, while 43 isolates were ST1791. The number of ST1791 isolates greatly exceeded that of the other ST isolates, and all ST1791 collections spanned the entire collection period (Table S1). No multiple ST1791 isolates were collected during any specific period. All the isolates in this study came from different patients and no repeated collections from the same patient occurred. Therefore, this study is the first to report the large-scale emergence of ST1791 in Anhui, China.

According to the growth curve, the growth rate and generation time of ST1791 were significantly higher than those of the other CC92 isolates in the experiment. ST1791 isolates exhibited a higher growth rate than other CC92 isolates under the same conditions, allowing them to reach their population peak earlier. Although the environmental conditions of the growth curve differed from those in the hospital and patient, the study findings were reasonably descriptive. The results of the *G. mellonella* infection model highlighted the threat posed by ST1791. Although the lethality rates of ST1791 and ST369 in G. *mellonella* were significantly higher than those of ST540, there were no significant differences between the other isolates and ST540, indicating that ST1791, like ST369, also exhibited strong virulence. Overall, ST1791 demonstrates strong growth ability and virulence, emerging as a new dangerous pathogen.

The capsular polysaccharide (CPS) of *A. baumannii* can resist dry environments and phagocytosis by host phagocytes, thereby enhancing the virulence of the strain (29). All ST1791 strains belonged to KL101, indicating consistency in the *cps* gene cluster. The differential genes and SNPs between ST1791 and other isolates were mainly concentrated in this gene cluster. A unique gene, *wbpI*, existed in the *cps* gene cluster of ST1791. The *wbpI* gene encodes UDP-2,3-diacetamido-2,3-dideoxy-d-glucuronate 2-epimerase, which catalyzes the epimerization of UDP-2,3-diacetamido-2,3-dideoxy-α-d-glucuronic acid (UDP-α-d-GlcNAc3NAcA) to UDP-2,3-diacetamido-2,3-dideoxy-α-d-mannuronic acid (UDP-α-d-ManNAc3NacA). The *wbpI* was first reported in *Pseudomonas aeruginosa* and *Bordetella pertussis* in 2007, where it synthesizes the O-antigen in lipopolysaccharides (30). The presence of *wbpI* in the *cps* gene cluster of ST1791-KL101 may suggest that the CPS and lipopolysaccharide composition of this strain is somewhat similar to that of these pathogenic bacteria and may enhance its virulence. In summary, the difference in virulence between ST1791 and other strains may mainly be attributable to variations in the *cps* gene cluster. However, whether KL101 enhances the virulence of *A. baumannii* requires further experimental verification. In addition to the differences in the *cps* gene cluster, ST1791 also carries six unique genes, including one tRNA-Asp (gtc) encoding gene, one glutathione S-transferase family protein-encoding gene and four genes with unknown function. The effects of these genes on the phenotype of ST1791 require further experimental investigation.

This study also identified a colistin-resistant ST1791 isolate, designated AB191, which arose via a *pmrB* mutation (c.899A > T p. Glu300Val). The combination of colistin resistance and high virulence results in the development of a clinically dangerous Gram-negative pathogen that requires further attention. Among the ST1791 isolates collected in this study, only AB191 was resistant to polymyxin, while it remained sensitive to tigecycline and minocycline. Additionally, most ST1791 isolates were sensitive to tigecycline and minocycline (Table S2). Therefore, a combined treatment of ST1791 with tigecycline and minocycline may help limit its spread.

In the NCBI database, ST195 and ST208 were the two most common *A. baumannii* types. Among the isolates in our collection, ST195 exhibited the greatest homology with ST1791, as reflected in the differences in SNPs. The average number of SNPs between our ST195 isolates and ST1791 isolates was approximately 1,200, which was lower than that observed within the ST1791 isolates. AB75 and AB82, which belong to ST208, were also highly similar to ST1791, and the average SNP difference between them and ST1791 was almost the same as that of ST195. In the phylogenetic tree constructed from the CC92 strain using NCBI data and ST1791 from this study, the ST1791 in this study, along with nine clinical ST1791 strains from Zhejiang in the NCBI database, formed a large branch. In contrast, the ST1791 strains in the NCBI database alone formed a small branch. The number of SNP differences between the ST791 strains in the NCBI database and the ST1791 isolates in this study was approximately 1,000, with some strains exhibiting as few as about 500 SNPs compared to the ST1791 isolates in this study. This indicates that ST1791 in this study has a high homology with ST1791 from Zhejiang. Furthermore, there are three other strains in this large branch that do not belong to ST1791: one ST1486 strain (17018) and two ST195 strains (2021CK-01384 and 2021CK-01552). The number of SNP differences between these two strains and the ST1791 strain in this study is less than 3,000. Therefore, ST1791 strains exhibit high homology with ST195 and ST1486 strains. Based on the isolates collected in this study, ST1791 has the highest homology with ST195, ST208, and ST1486. However, to elucidate the evolutionary relationship between ST1791 and other strains, more *A. baumannii* isolates from Anhui, China, must be collected for more in-depth research.

This study has certain limitations. Regardless of whether Snippy or BacWGSTdb is used, both can only identify SNPs. If there are significant sequence differences in the genome, neither tool can effectively identify them. Roary, which identifies gene differences, relies on an annotated reference genome. If some genes are not annotated in the reference genome, Roary cannot identify them. Consequently, many unique regions in the ST1791 genome may remain undetected. Additionally, the number of strains collected was insufficient, resulting in a certain degree of contingency. Similarly, our isolate collection strategy had some limitations, as it only included isolates from a single hospital. There might be other isolates with higher homology to ST1791 than ST195 and ST208.

In conclusion, this study reported a large-scale emergence of *A. baumannii* clone ST1791 in Anhui, China, which exhibited significant drug resistance, a high growth rate, and strong virulence. The emergence of ST1791 *A. baumannii* poses a substantial threat to public health. Therefore, enhanced surveillance and strict infection control strategies are urgently needed to limit its further spread.

## MATERIALS AND METHODS

### Bacterial strains and growth conditions

From November 2021 to April 2022, a total of 132 non-repeating MDR *A. baumannii* isolates were isolated from clinical samples of patients at a tertiary-grade hospital in Hefei, Anhui Province (Table S1). The VITEK 2 Compact system (bioMérieux, France) and the VITEK MS IND MALDI TOF time-of-flight mass spectrometry system (bioMérieux, France) were used to identify the strain species and perform antibiotic susceptibility testing. The antibiotics included in the susceptibility tests were piperacillin/tazobactam, ticarcillin/clavulanic acid, ceftazidime, cefepime, imipenem, meropenem, polymyxin B, tobramycin, doxycycline, minocycline, ciprofloxacin, levofloxacin, trimethoprim/sulfamethoxazole, tigecycline, and cefoperazone/sulbactam.

### Plasmid construction

We designed primers incorporating 15 bp homologous sequences of plasmid pUCK19 (26) to amplify the target gene sequence. The amplified product was then ligated into pUCK19, which had been digested with *Bam*HI and *Sal*I, using T4 ligase (Table 2). The recombinant plasmid was transformed into competent *Escherichia coli* Top 10 by heat shock transformation. Subsequently, plasmids were extracted from the Top 10 cells, and the recombinant plasmids were then transformed into *A. baumannii* AB60 competent cells by electroporation. The transformed cells were stored at −80°C until further use.

**TABLE 2:** Plasmids and primers used in this study

Plasmids or primers	Description or oligonucleotide (5′−3′)^*a*^	Source or application
Plasmids		
pUCK19	Transformed by inserting a kanamycin resistance gene into pUC19	Y. Huang et al., 2023 (26)
pUCK19-*pmrB*-AB60	pUCK19 derivative, for *pmrB* in strain AB60, Amp^r^ Kan^r^	This study
pUCK19-*pmrB*-AB191	pUCK19 derivative, for *pmrB* mutation in strain AB191, Amp^r^ Kan^r^	This study
Primers		
*cpn60*-F	GGTGCTCAACTTGTTCGTGA	MLST (Oxford scheme)
*cpn60*-R	CACCGAAACCAGGAGCTTTA	MLST (Oxford scheme)
*gdhB*-F	GCTACTTTTATGCAACAGAGCC	MLST (Oxford scheme)
*gdhB*-R	GTTGAGTTGGCGTATGTTGTGC	MLST (Oxford scheme)
*gltA*-F	AATTTACAGTGGCACATTAGGTCCC	MLST (Oxford scheme)
*gltA*-R	GCAGAGATACCAGCAGAGATACACG	MLST (Oxford scheme)
*gpi*-F	GAAATTTCCGGAGCTCACAA	MLST (Oxford scheme)
*gpi*-R	TCAGGAGCAATACCCCACTC	MLST (Oxford scheme)
*gyrB*-F	TGAAGGCGGCTTATCTGAGT	MLST (Oxford scheme)
*gyrB*-R	GCTGGGTCTTTTTCCTGACA	MLST (Oxford scheme)
*recA*-F	CCTGAATCTTCYGGTAAAAC	MLST (Oxford scheme)
*recA*-R	GTTTCTGGGCTGCCAAACATTAC	MLST (Oxford scheme)
*rpoD*-F	ACCCGTGAAGGTGAAATCAG	MLST (Oxford scheme)
*rpoD*-R	TTCAGCTGGAGCTTTAGCAAT	MLST (Oxford scheme)
AB-*pmrB*-u-pUCK19-*Sal*I	CGCGTCGACttaattccacttatgactcacc	Amplification of the *pmrB* gene of *A. baumannii*
AB-*pmrB*-d-pUCK19-*Bam*HI	CGCGGATCCtcacactcttgtttcatgtaa	Amplification of the *pmrB* gene of *A. baumannii*

^*a*^ Amp^r^, ampicillin resistant; Kan^r^, kanamycin resistant.

### Determination of MIC of polymyxin B by broth microdilution method

Polymyxin B MIC values were determined for different strains using a previously described method in accordance with the Clinical and Laboratory Standards Institute guidelines (31, 32). Briefly, Mueller-Hinton (MH) broth was used to prepare polymyxin B at different concentration gradients, and 75 µL was dispensed into each well of a 96-well plate. Several monoclonal strains were selected, and the bacterial concentration was adjusted in MH medium to an optical density (OD_600_) of 0.4. This suspension then diluted 200-fold in an appropriate volume of MH medium. Next, 75 µL of the diluted suspension was inoculated into each well of the 96-well plate containing MH medium with different concentrations of polymyxin B, resulting in a total volume of 150 µL per well. The plate was incubated at 37°C with shaking at 220 rpm. After 24 hours, the plate was taken out and photographed, and the MIC values were recorded.

### Growth curves

The growth curves of *A. baumannii* were established manually in Luria-Bertani broth (LB) medium. Overnight cultures were diluted to an optical density (OD_600_) of 0.02 in LB medium and incubated in 96-well plates at 37°C with shaking at 220 rpm. The OD_600_ was measured every 30 minutes using a microplate reader until the growth curves reached their peak and stabilized.

To model the growth dynamics, a mathematical model was constructed using the Growthcurver R package (33). The logistic equation describes the relationship between the population size *N_t_* and time *t* as follows:


$$N_{t}=\frac{K}{1+\left(\frac{K-N_{0}}{N_{0}}\right)e^{-rt}}$$

where *N_0_* represents the initial population size, *K* represents the maximum population size in a given environment, and *r* represents the growth rate without any limitations on the population.

### *G. mellonella* infection model

The virulence of *A. baumannii* was assessed using the *G. mellonella* infection model, as previously described (34). Larvae (0.3–0.4 g) were kept protected from light and used within 3 days after shipment (Tianjin Huiyude Biotechnology Co., Ltd.). Prior to injection, the bacterial pellet was washed with sterile saline and diluted to 1 × 10^8^ CFU/mL. Using a 1 mL insulin syringe (Shanghai Kindly Ent Dev), 10 µL of the bacterial suspension was injected into the center of the larval second gastropod. A group of 10 larvae was randomly selected for injection. Each treatment was performed in triplicate, using a total of 30 larvae. After injection, the larvae were incubated at 37°C, and their survival was monitored daily for 3 days. Larvae were considered dead when they no longer responded to touch. Negative controls consisted of larvae that were either uninjected or injected with 10 µL of sterile saline. In all cases, no deaths occurred in the negative control groups.

### Whole-genome sequencing, assembly, and annotation

Second-generation sequencing was performed using Illumina HiSeq 4000 platform (Nuosai Jiyin Zu Research Center Limited Company, Beijing, China). Quality filtering of the raw sequencing data was carried out using fastp (35). The filtered data were then assembled using Unicycler v0.4.8 (36), and the assembled genome was annotated using the Prokka 1.14.6 (37) (Table S6).

### Genome profiling and comparative genomics analysis

All clinical *A. baumannii* genomes (including whole-genome sequencing and whole-genome shotgun sequencing data) available in the NCBI Pathogen Detection database (https://www.ncbi.nlm.nih.gov/pathogens/isolates/#acinetobacter%20baumannii; under “Filters,” click on “clinical” in “Isolation Type”) were downloaded for comparative genomics as of 31 December 2022. Acquired antimicrobial resistance genes and virulence genes were identified by aligning genome sequences with the NCBI and Virulence Factor Database (VFDB) using ABRicate 1.0.1 (https://github.com/tseemann/abricate). MLST analysis of isolates was performed using MLST 2.1 (https://github.com/tseemann/mlst) (38). Capsular typing of the isolates was conducted using Kaptive v2.0.0 (39, 40). Comparative genomic analysis of different isolates was carried out using HarvestTools kit (Parsnp, Gingr, and HarvestTools), and phylogenetic trees were constructed using Parsnp 1.7.4 based on SNPs of the core genome using the maximum likelihood method (recombinant regions in the genome were not deleted) (41). We used Interactive Tree of Life v5 (http://itol.embl.de/) to construct phylogenetic trees (42). Snippy 4.6.0 (https://github.com/tseemann/snippy) and BacWGSTdb (http://bacdb.cn/BacWGSTdb/analysis_multiple.php) were employed to detect SNPs and mutations between isolates without deleting recombination regions in the genome (43). BacWGSTdb was utilized to quickly count the number of SNPs among different isolates, while Snippy was used to analyze the specific locations of SNPs in the genomes of the isolates. Roary was employed to analyze gene differences between isolates (44). The commands used for each software can be found in Table S6. Easyfig 2.2.5 was used to visualize the sequence alignments (25), and ComplexHeatmap R package was used to create heatmaps (45).

### Statistical analyses

All analyses were performed using Prism software (GraphPad Software, La Jolla, CA, USA) and R scripts. Differences between groups were analyzed using two-sample Wilcoxon tests. Error bars represent SEM. All experiments were repeated at least three times. Signiﬁcance was deﬁned as *P < 0.05; **P < 0.01; ***P < 0.001; and ****P < 0.0001.

## Data Availability

The whole genome sequences generated in the current study are available in the NCBI database (BioProject: PRJNA917695).

## SUPPLEMENTAL MATERIAL

Supplemental figures aac.01262-24-s0001.pdf Figures S1 to 12.

Figure S13 aac.01262-24-s0002.pdf Circular phylogenetic tree constructed by CC92 strains from the NCBI database and ST1791 isolates collected in this study.

Figure S14 aac.01262-24-s0003.pdf Circular phylogenetic tree constructed by the screened 5,795 CC92 strains from the NCBI database and the ST1791 isolates collected in this study.

Figure S15 aac.01262-24-s0004.pdf The position of the ST1791 strains in the phylogenetic tree.

Table S1 aac.01262-24-s0005.xlsx The basic information of the patients with the isolates collected in this study.

Table S2 aac.01262-24-s0006.xlsx Minimum inhibitory concentration of different antibiotics on isolates (CLSI).

Table S3 aac.01262-24-s0007.xlsx Basic characteristics of the genomes of the strains collected in this study.

Table S4 aac.01262-24-s0008.xlsx Unique genes carried by ST1791 identified by Roary.

Table S5 aac.01262-24-s0009.xlsx Number of SNPs among different isolates.

Table S6 aac.01262-24-s0010.xlsx The commands used in this study.

ASM does not own the copyrights to Supplemental Material that may be linked to, or accessed through, an article. The authors have granted ASM a non-exclusive, world-wide license to publish the Supplemental Material files. Please contact the corresponding author directly for reuse.
