# Supplementary material for: Characterization and genomic analysis of the highly virulent Acinetobacter baumannii ST1791 strain dominating in Anhui, China
Source: Antimicrob Agents Chemother. 2024 Dec 6;69(1):e01262-24. doi: 10.1128/aac.01262-24 (PMC11784083; doi:10.1128/aac.01262-24)
Supplement: Supplemental figures — Figures S1 to 12. [file aac.01262-24-s0001.pdf]

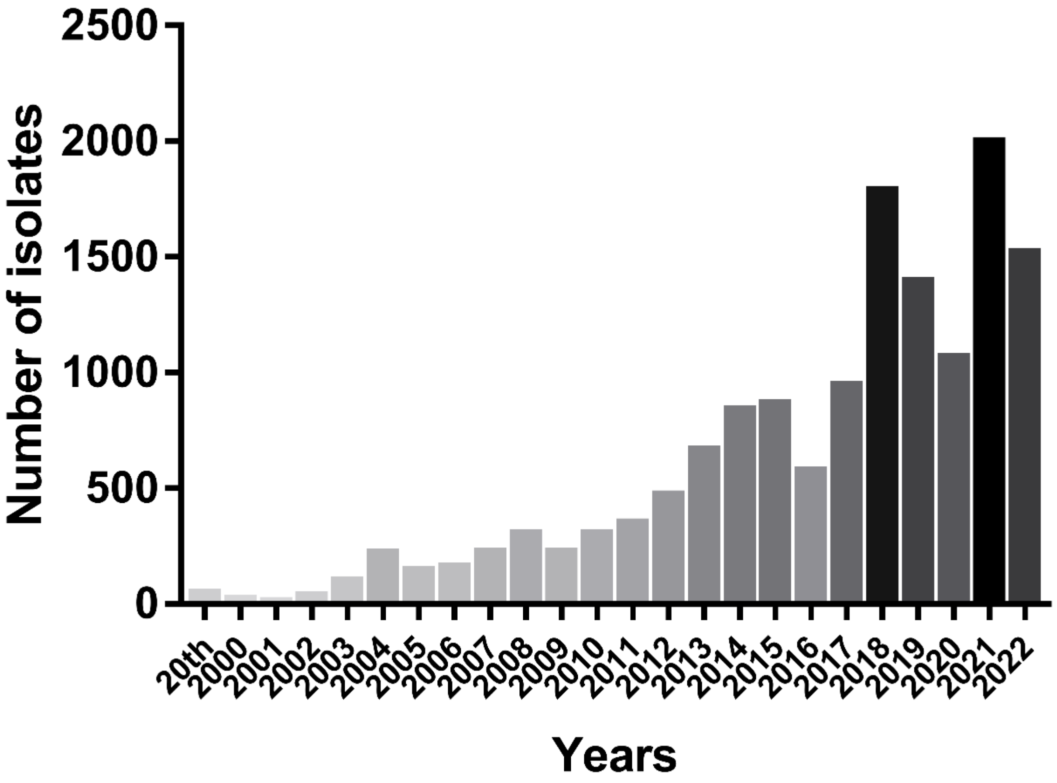

Fig. S1 Isolation year distribution of clinical *Acinetobacter baumannii* isolates in NCBI database.

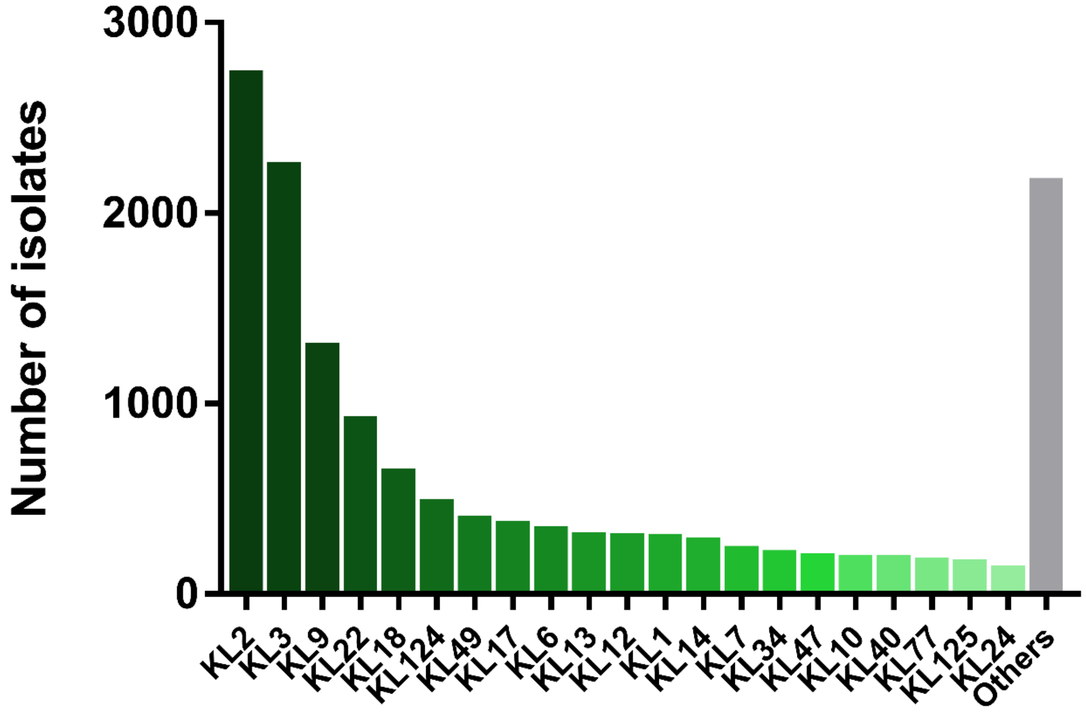

Fig. S2 Capsular type (K-type) distribution of clinical isolates of *A. baumannii* in NCBI database

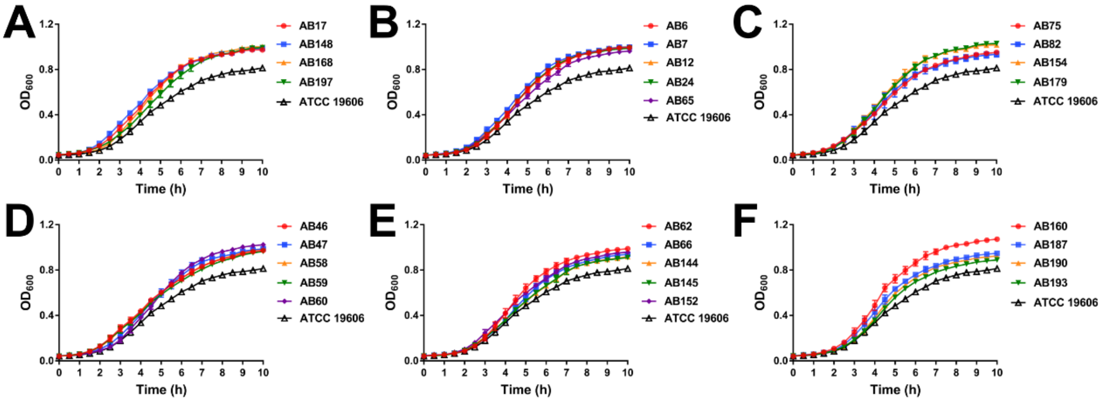

Fig. S3 Growth curves of collected ST195, ST208, and ST369 isolates.

(A) is the growth curves of ST195 isolates. (B) and (C) are the growth curves of ST208 isolates. (D), (E), and (F) are the growth curves of ST369 isolates.

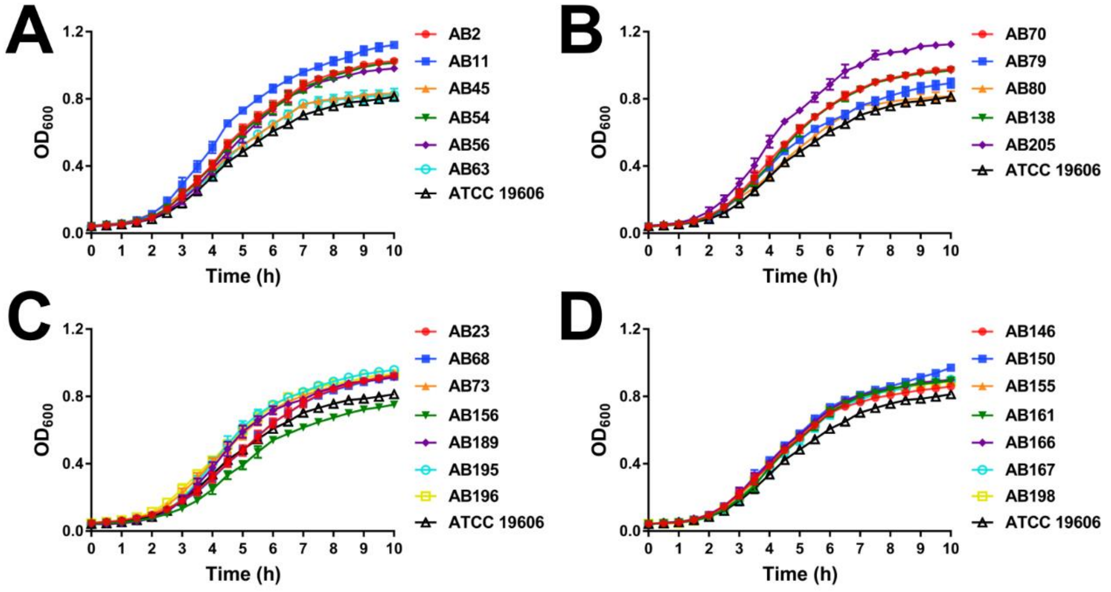

Fig. S4 Growth curves of the collected ST540, ST1968, and ST2499 isolates.

(A) and (B) are the growth curves of ST540 isolates. (C) is the growth curves of ST1968 isolates. (D) is the growth curves of ST2499 isolates.

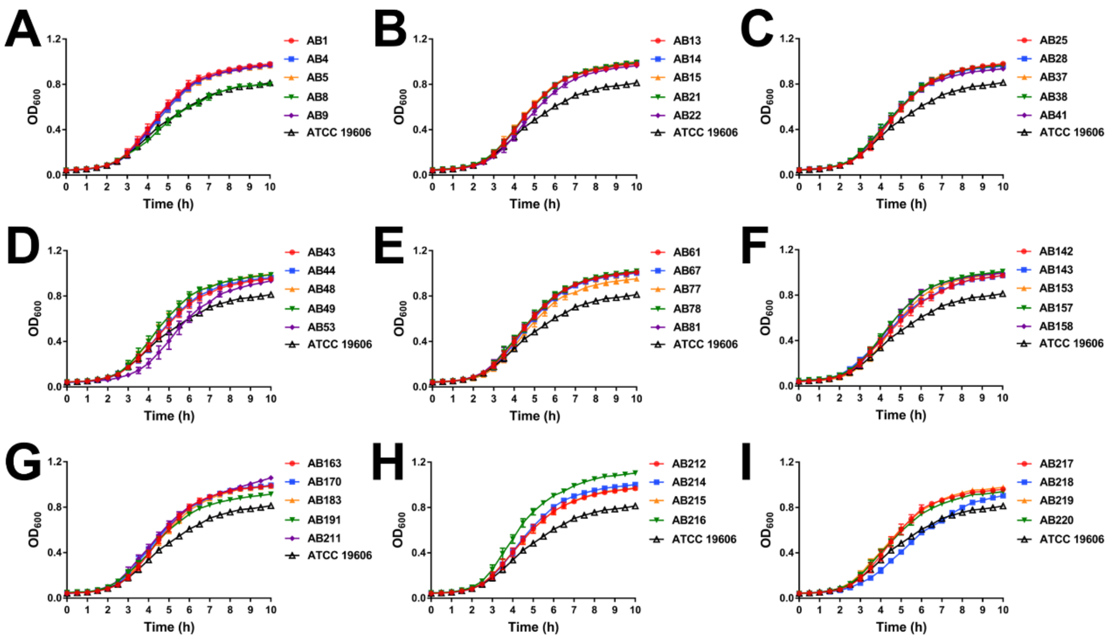

Fig. S5 Growth curves of the collected ST1791 isolates.

(A), (B), (C), (D), (E), (F), (G), (H), and (I) are the growth curves of ST1791 isolates.

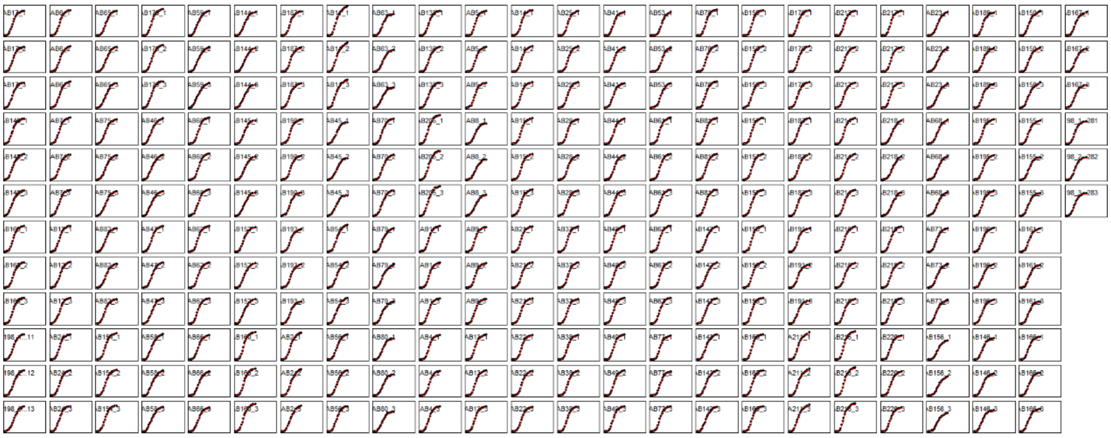

Fig. S6 The growth curve model of each isolate simulated using the Growthcurver R package.

The names of the different isolates are marked in the upper left corner of the growth curves.

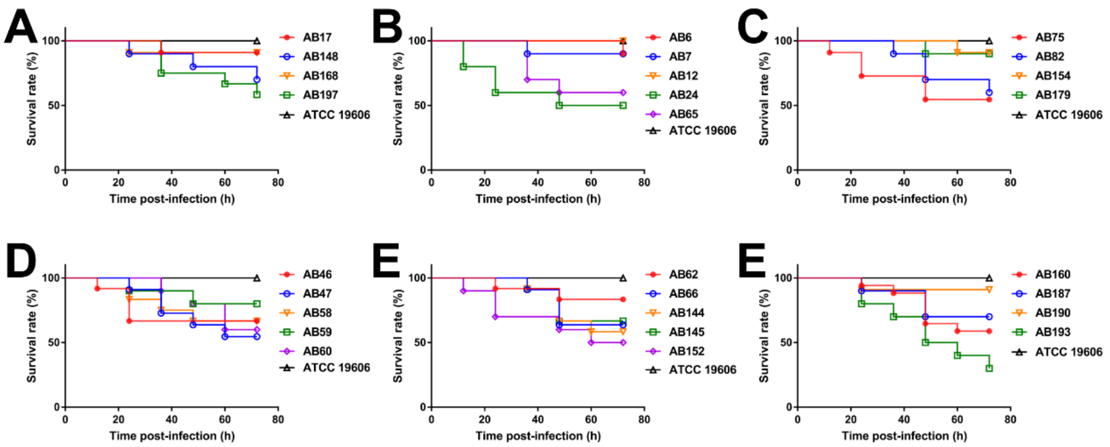

Fig. S7 *Galleria mellonella* infection model assay of ST195, ST208, and ST369 isolates. (A) is the *G. mellonella* infection model assay result of ST195 isolates. (B) and (C) are the *G. mellonella* infection model assay results of ST208 isolates. (D), (E), and (F) are the *G. mellonella* infection model assay results of ST369 isolates.

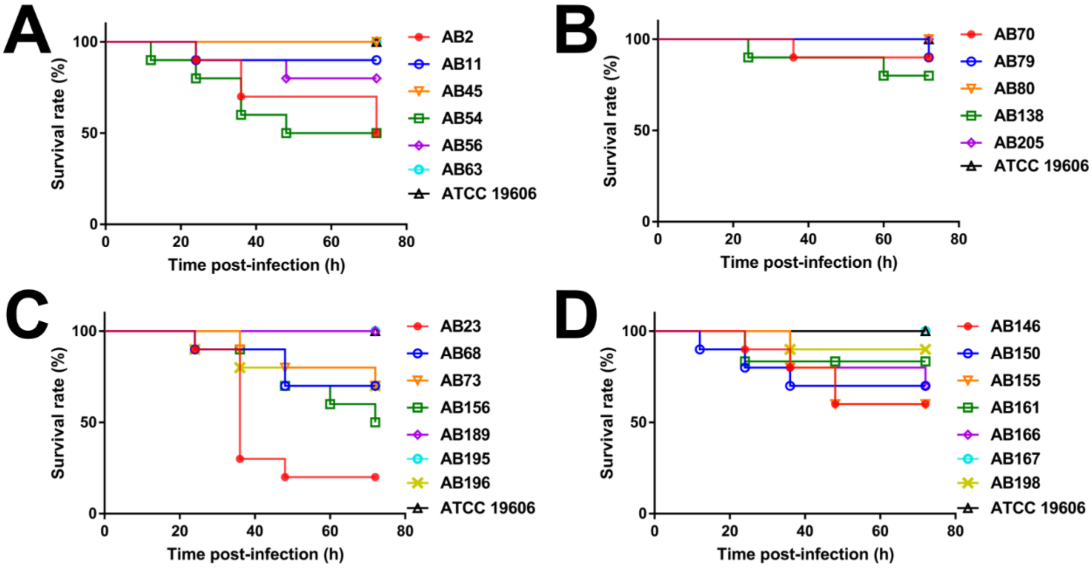

Fig. S8 *G. mellonella* infection model assay of the collected ST540, ST1968, and

ST2499 isolates.

(A) and (B) are the *G. mellonella* infection model assay results of ST540 isolates. (C) is the *G. mellonella* infection model assay result of ST1968 isolates. (D) is the *G. mellonella* infection model assay result of ST2499 isolates.

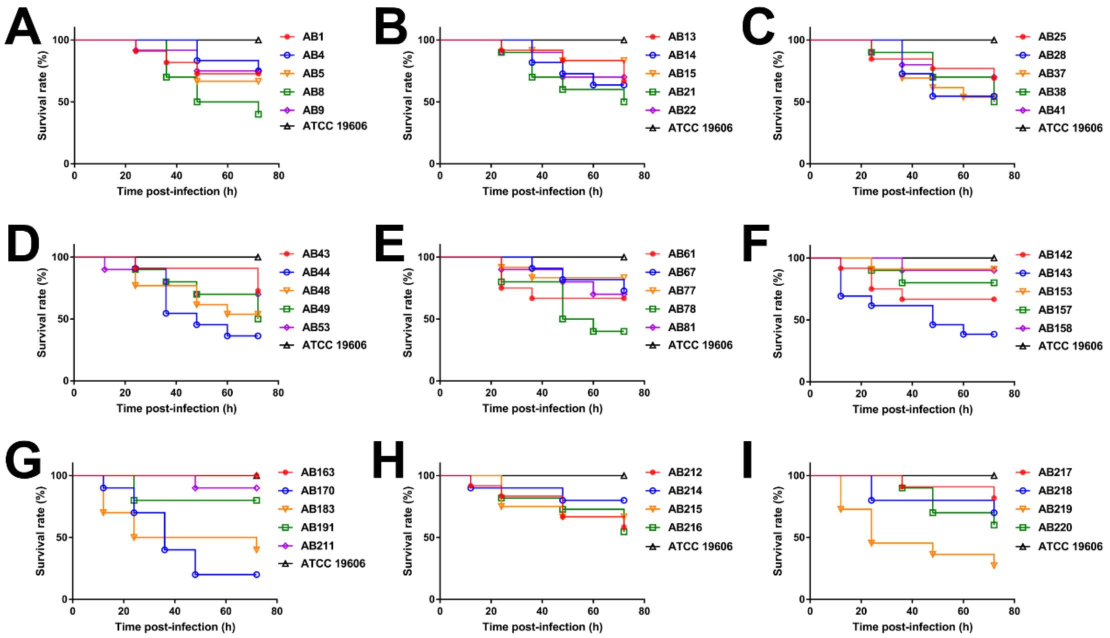

Fig. S9 *G. mellonella* infection model assay of the collected ST1791 isolates.

(A), (B), (C), (D), (E), (F), (G), (H), and (I) are the *G. mellonella* infection model assay results of ST1791 isolates.

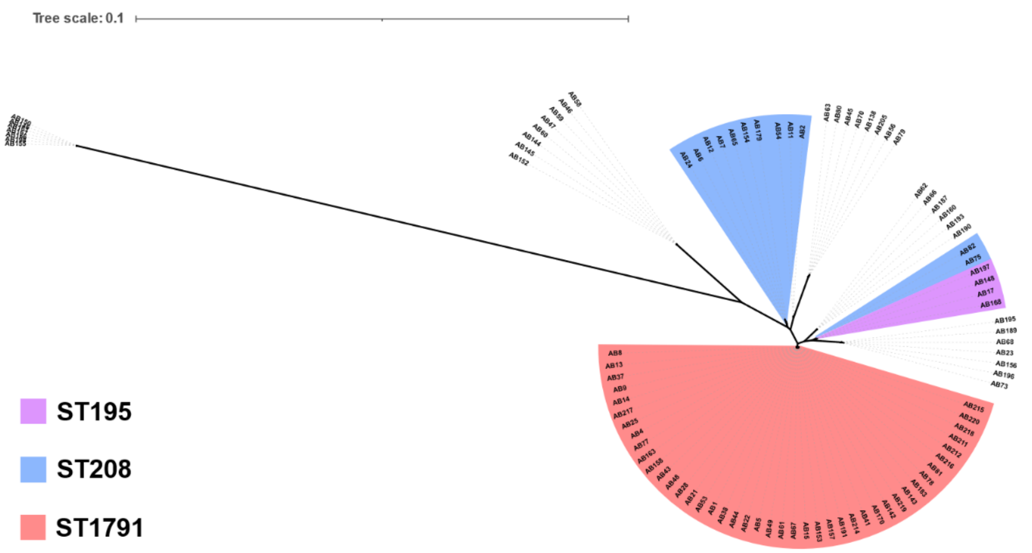

Fig. S10 Unrooted phylogenetic tree of isolates collected in this study.

The unrooted phylogenetic tree constructed using the ATCC 19606 genome as the reference genome. Purple, blue and red represent ST195, ST208 and ST1791 respectively.

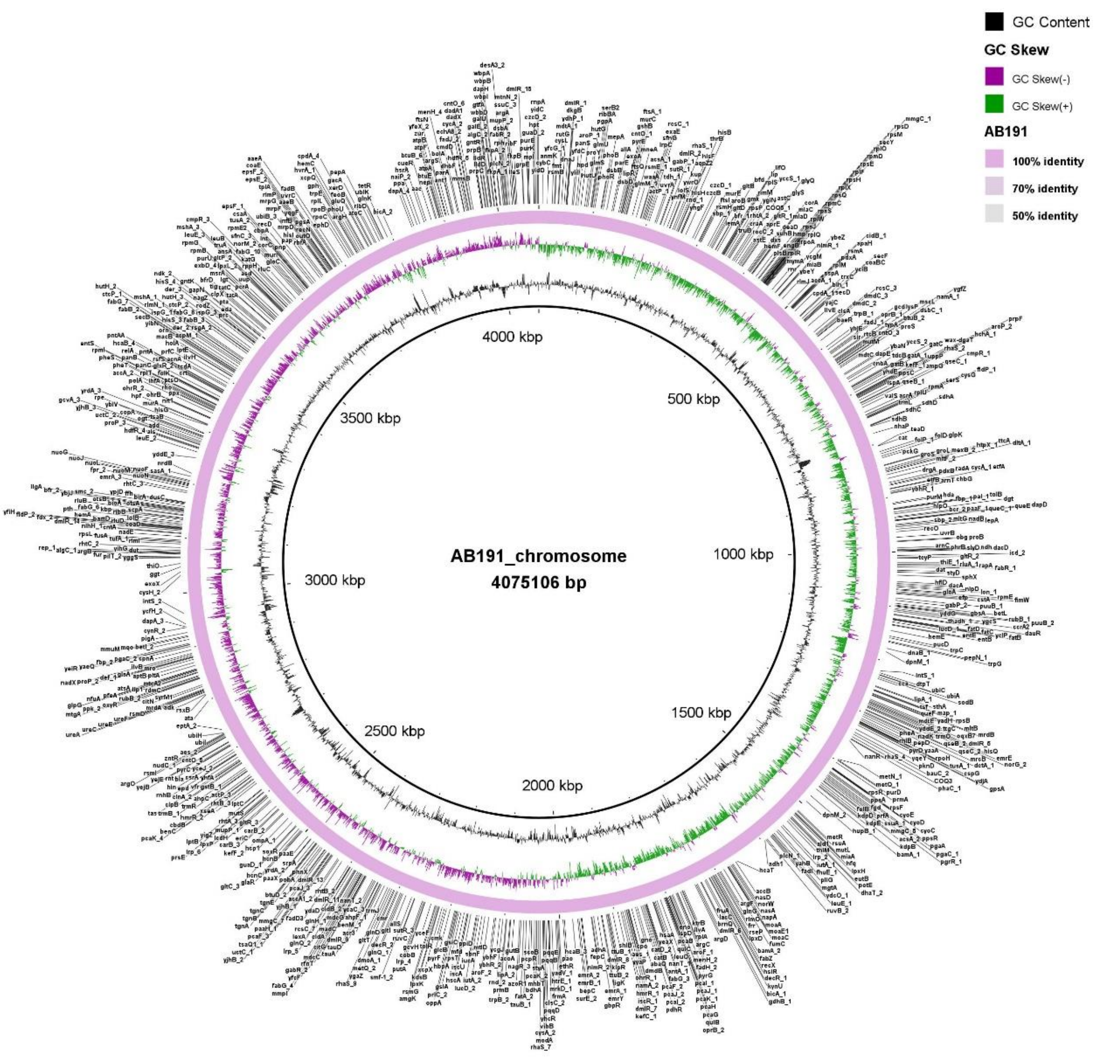

Fig. S11 Circular chromosome map of *A. baumannii* AB191 (accession no. CP166770).

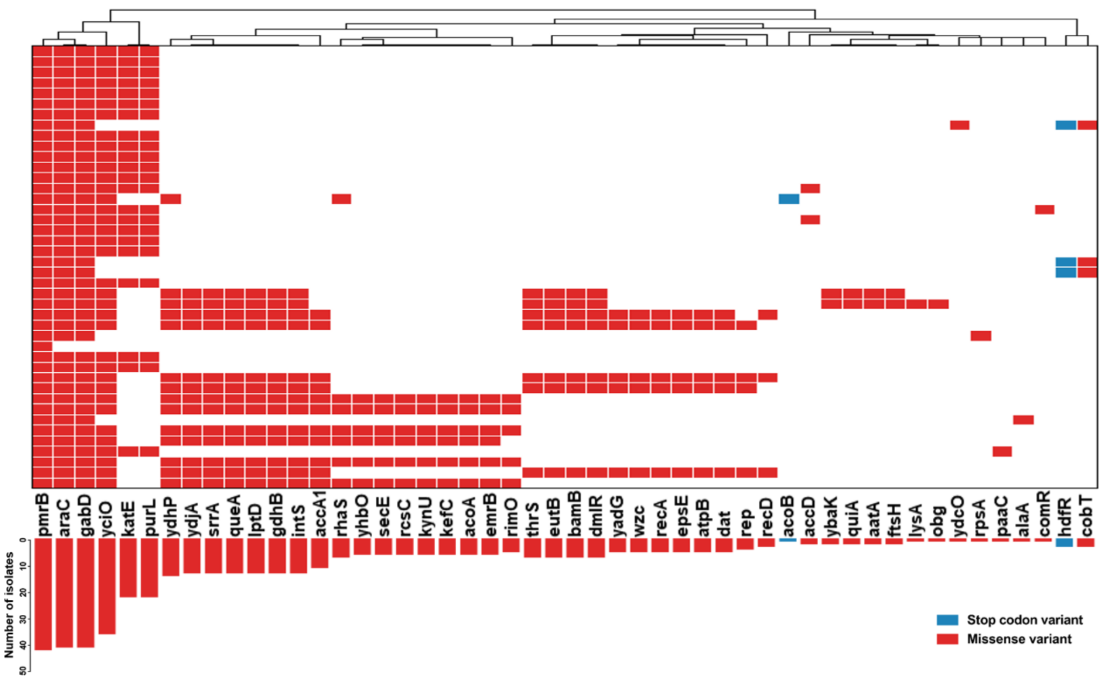

Fig. S12 Mutations in polymyxin-related genes in ST1791 *A. baumannii* isolates.

The reference genome is the AB191 genome. Missense variants are shown in red and stop codon variants are shown in blue.
