# Supplementary material for: Characterization and genomic analysis of the highly virulent Acinetobacter baumannii ST1791 strain dominating in Anhui, China
Source: Antimicrob Agents Chemother. 2024 Dec 6;69(1):e01262-24. doi: 10.1128/aac.01262-24 (PMC11784083; doi:10.1128/aac.01262-24)
Supplement: Figure S13 — Circular phylogenetic tree constructed by CC92 strains from the NCBI database and ST1791 isolates collected in this study. [file aac.01262-24-s0002.pdf]

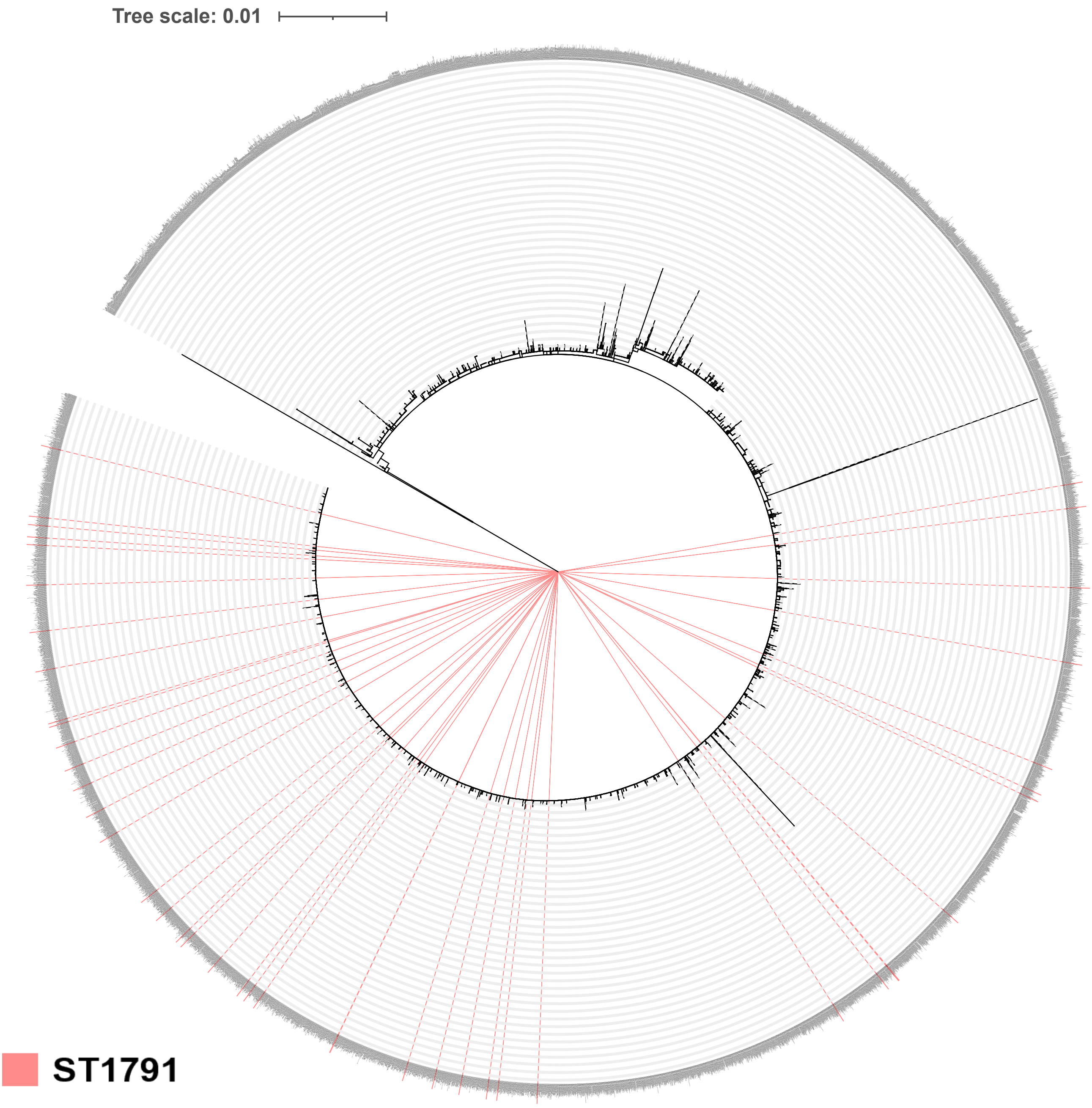

Fig. S13 Circular phylogenetic tree constructed by CC92 strains from the NCBI database and ST1791 isolates collected in this study.

The circular phylogenetic tree constructed using the ATCC 19606 genome as the reference genome. Red represents ST1791 isolates.
