# Supplementary material for: Characterization and genomic analysis of the highly virulent Acinetobacter baumannii ST1791 strain dominating in Anhui, China
Source: Antimicrob Agents Chemother. 2024 Dec 6;69(1):e01262-24. doi: 10.1128/aac.01262-24 (PMC11784083; doi:10.1128/aac.01262-24)
Supplement: Figure S14 — Circular phylogenetic tree constructed by the screened 5,795 CC92 strains from the NCBI database and the ST1791 isolates collected in this study. [file aac.01262-24-s0003.pdf]

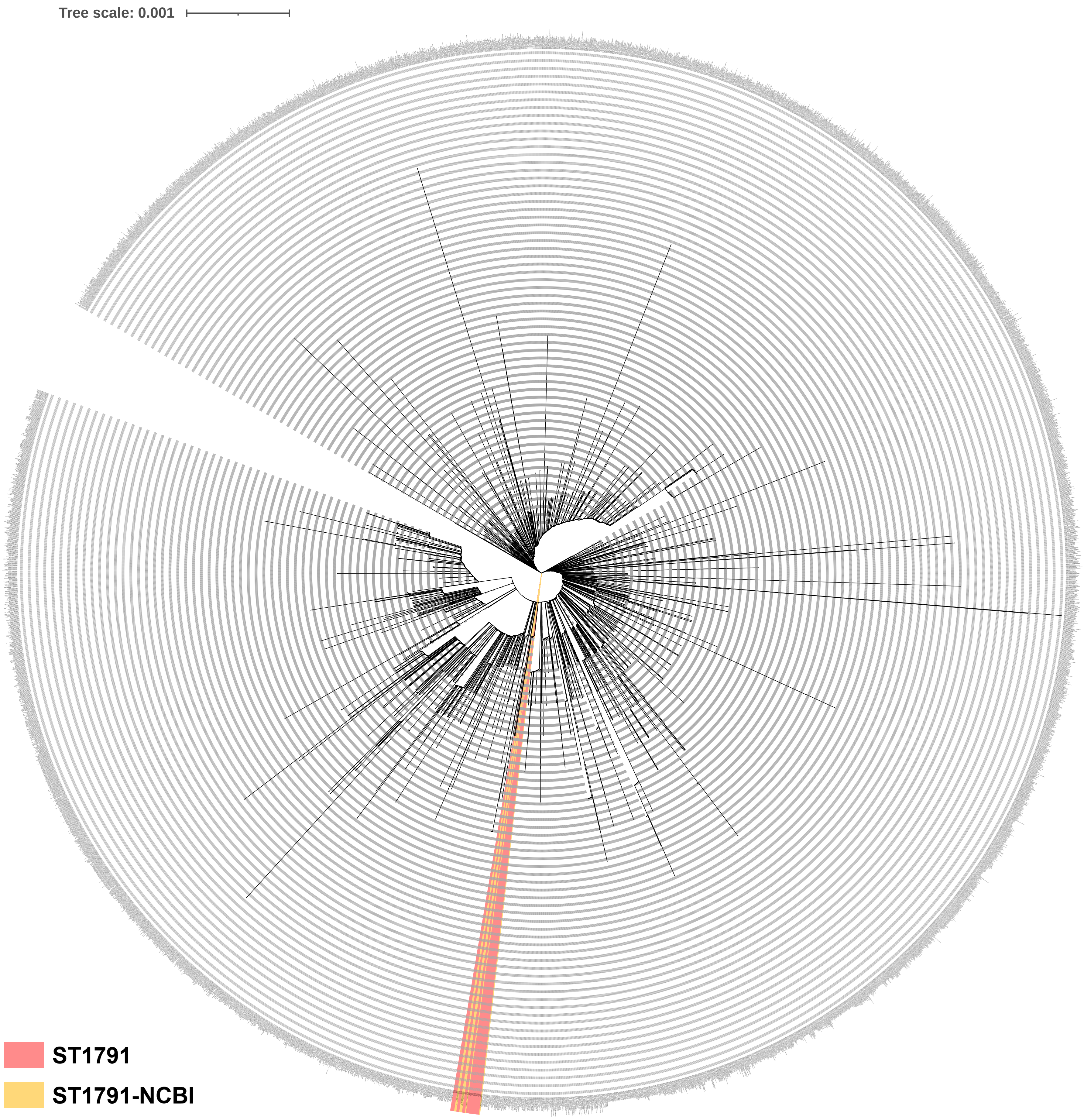

Fig.S14 Circular phylogenetic tree constructed by the screened 5,795 CC92 strains from the NCBI database and theST1791 iso lates collected in this study.

The circular phylogenetic tree constructed using BM2333 (ST208, CP091328) genome as the reference genome. Red represents ST1791 isolates collected in this study and orange represents ST1791 strains from the NCBI database.
