# Supplementary material for: Characterization and genomic analysis of the highly virulent Acinetobacter baumannii ST1791 strain dominating in Anhui, China
Source: Antimicrob Agents Chemother. 2024 Dec 6;69(1):e01262-24. doi: 10.1128/aac.01262-24 (PMC11784083; doi:10.1128/aac.01262-24)
Supplement: Figure S15 — The position of the ST1791 strains in the phylogenetic tree. [file aac.01262-24-s0004.pdf]

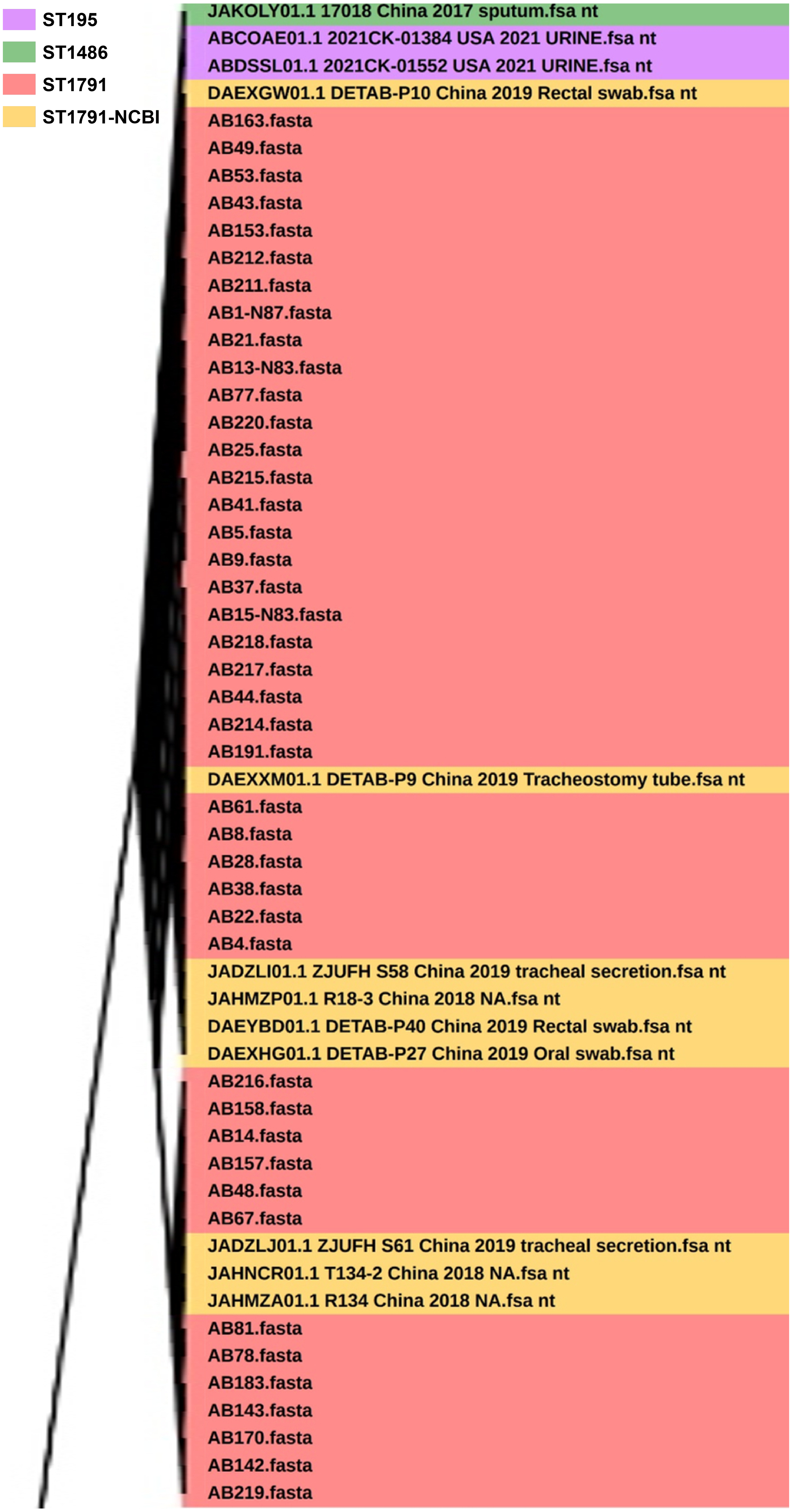

Fig.S15 The position of the ST1791 strains in the phylogenetic tree.

See **Fig. S14** for details of the phylogenetic tree. Purple represents the ST195 strains, green represents the ST1486 strain, red represents the ST1791 isolates collected in this study, and orange represents the ST1791 strains from the NCBI database.
